# ESE-1/ELF3 mRNA expression associates with poor survival outcomes in HER2^+^ breast cancer patients and is critical for tumorigenesis in HER2^+^ breast cancer cells

**DOI:** 10.18632/oncotarget.18710

**Published:** 2017-06-27

**Authors:** Adwitiya Kar, Arthur Gutierrez-Hartmann

**Affiliations:** ^1^ Cancer Biology Training Program, University of Colorado Anschutz Medical Campus, Aurora, CO 80045, USA; ^2^ Department of Medicine, University of Colorado Anschutz Medical Campus, Aurora, CO 80045, USA; ^3^ Department of Biochemistry & Molecular Genetics, University of Colorado Anschutz Medical Campus, Aurora, CO 80045, USA; ^4^ Program in Molecular Biology, University of Colorado Anschutz Medical Campus, Aurora, CO 80045, USA

**Keywords:** SKBR3, luminal B, xenograft, Akt, mTOR

## Abstract

ESE-1/Elf3 and HER2 appear to establish a positive feedback regulatory loop, but the precise role of ESE-1 in HER2^+^ breast tumorigenesis remains unknown. Analyzing public repositories, we found that luminal B and HER2 subtype patients with high *ESE-1* mRNA levels displayed worse relapse free survival. We stably knocked down ESE-1 in HER2^+^ luminal B BT474 cells and HER2 subtype SKBR3 cells, which resulted in decreased cell proliferation, colony formation, and anchorage-independent growth *in vitro*. Stable ESE-1 knockdown inhibited HER2-dependent signaling in BT474 cells and inhibited mTOR activation in SKBR3 cells, but reduced Akt signaling in both cell types. Expression of a constitutively-active Myr-Akt partially rescued the anti-proliferative effect of ESE-1 knockdown in both cell lines. Furthermore, ESE-1 knockdown inhibited cyclin D1, resulting in a G1 delay in both cell lines. Finally, ESE-1 knockdown completely inhibited BT474 cell xenograft tumors in NOD/SCID female mice, which correlated with reduced *in vitro* tumorsphere formation. Taken together, these results reveal the ESE-1 controls transformation via distinct upstream signaling mechanisms in SKBR3 and BT474 cells, which ultimately impinge on Akt and cyclin D1 in both cell types to regulate cell proliferation. Particularly significant is that ESE-1 controls tumorigenesis and is associated with worse clinical outcomes in HER2 breast cancer.

## INTRODUCTION

The ETS transcription factor family consists of 27 ETS genes in humans. They can be structurally categorized into 11 subfamilies (ETS, ERG, GABPA, ELF, ESE, ERF, TEL, PEA3, SPI, TCF and PDEF) [1–3]. In the biology of cancer, ETS proteins are involved almost in every known mechanism of tumorigenesis, which includes overexpression, gene fusion, sub-cellular localization, activator to repressor switch, and post translational modifications [4]. Most of the ETS proteins respond to mitogenic growth factors and in turn act as transcriptional regulators of genes involved in cell cycle progression, invasion and metastasis [4, 5]. High mRNA expression of Pdef, Pea3, Ese-1, Ese-2, Tel, and Nerf has been reported in epithelial cell compartments of mammary tumors [6]. Of these, ESE-1/ELF3 and ESE-2/Elf5 of the ESE sub-family are well documented for their role in mammary tumorigenesis [7–10].

ESE-1 (epithelial specific ETS factor- 1) is a 42 kDa protein [4] and mRNA expression of ESE-1 has been recorded in several human and rodent epithelial tissues [11–14]. In humans, the ESE-1 gene locus maps to chromosome 1q31.1, and it is located in a region that is commonly amplified in breast cancer and is often overexpressed early during human breast tumorigenesis [15, 16]. The ESE-1 protein is characterized by a conserved winged helix turn helix DNA binding domain known as the ETS domain, a transcription activation domain or TAD, a conserved pointed domain for protein-protein interactions, and an unique serine aspartate rich region or the SAR domain [2]. We have previously established that stable expression of HA-ESE-1 or GFP-ESE-1 fusions in the ESE-1 negative, benign MCF-10A and MCF-12A human mammary cell lines impose increased cellular proliferation, migration, invasion and colony number in soft agar [7, 17]. We reported that ESE-1 alone initiates this transformation via cytoplasmic localization of its autonomously functioning 40 amino acid SAR domain [7, 18]. We also reported that ESE-1 controls proliferation to control transformation in the luminal A cell lines MCF7 and ZR75-1. In this paper, we investigate the scope and the extent to which ESE-1 controls malignant properties such as proliferation, colony formation ability, anchorage independent growth, and *in vivo* tumorigenesis in HER2^+^ tumorigenic BT474 and SKBR3 cell lines.

Over-expression of *ESE-1* mRNA directly correlates with *HER2*^*+*^ expression in human breast cancers, and *ESE-1* mRNA over-expression is often detected in human breast ductal carcinoma *in situ*, an early stage cancer that also over-expresses HER2 [16, 19]. Furthermore, a positive feedback loop appears to exist between the *HER2* proto-oncogene and *ESE-1*, whereby HER2 signaling induces *ESE-1* gene expression, and nuclear ESE-1 trans-activates the *HER2* promoter [20, 21]. In HER2^+^ SKBR3 breast cancer cells, disruption of ESE-1/Sur2 interaction with pharmacological inhibitors attenuates HER2-dependent signaling, at 72 hours [22]. But given the fact that Sur2 is a mediator protein commonly employed by the Pol II transcriptional machinery and that the small molecule inhibitor caused apoptosis (which is not observed with ESE-1 knockdown in transformed cell lines), the specific role of ESE-1 in the transformative process was not clear. Also, to date there have been no studies elucidating the prognostic value of ESE-1 expression or the mechanisms underlying ESE-1 mediated transformation in HER2^+^ breast cancers *in vitro* and *in vivo*. Using two subtype specific cell lines, HER2^+^ luminal B BT474 and ER^-^/PR^-^ HER2 subtype SKBR3 we report that the relationship between ESE-1 and HER2, though exists is more complicated than previously appreciated. We show that persistent knockdown of ESE-1 inhibits HER2 and mTOR expression in a cell type specific and temporal manner attenuating downstream pAkt, pGSK3β, phospho-p70S6K and cyclin D1, and in turn causing a delay in G1 progression. ESE-1 knockdown significantly inhibits tumorsphere formation and tumorigenesis *in vivo*. Clinically, high ESE-1 expression associates with lower survival outcomes in the luminal B and HER2 subtype. Taken together, these data establish that ESE-1 plays an important role in HER2 tumorigenesis and has the potential to serve as a prognostic marker in HER2^+^ breast cancer patients.

## RESULTS

### Analysis of ESE-1 copy number and mRNA expression in breast cancer tumor types and cell lines

**Figure 1:**
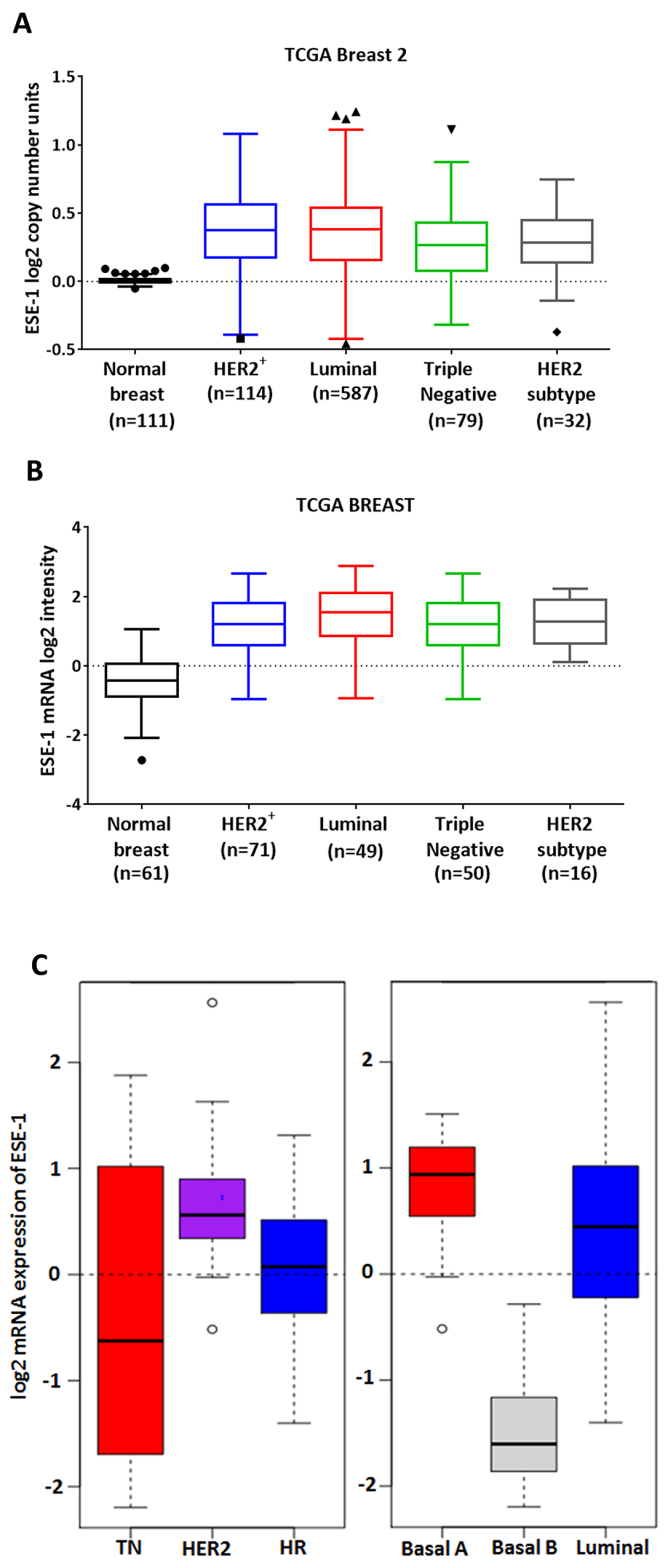
*ESE-1* mRNA expression in breast cancer tissues and cell lines **(A)** A Tukeys box plot using Graphpad Prism shows the copy number variations of ESE-1/ELF3 gene in all patient subtypes in the TCGA Breast 2 cohort (log2 ratios cancer versus normal) from Oncomine. The range of the box is the inter quartile range for each tissue type. Anything above 3IQR is shown as outliers as solid triangles and squares. All subtypes bear an elevated ESE-1 DNA copy number compared to the control (Anova P value < 0.0001). **(B)** Tumor and normal breast tissue gene expression was obtained following array normalization by processing the TCGA Breast dataset through Oncomine (www.oncomine.org). A Tukeys boxplot showing that ESE-1 mRNA level (log2 median centered intensities obtained from microarray) is upregulated in the different subtypes of breast carcinomas (Anova P value <.0001) compared to the normal breast. The range of the box is the inter quartile range for each tissue type. High *ESE-1* mRNA expression and ESE-1 protein nuclear localization in HER2^+^ cells. **(C)** Box plot of gene expression for ESE-1/ELF3 across cell lines grouped into clinical subtypes based on the annotation data from Neve et al using GOBO. The range of the box is the inter-quartile range for each tumor type. Anything above 3IQR are shown as outliers, and represented as circles. *ESE-1* gene expression is high in the HER2 enriched types.

Since the *ESE-1* gene locus maps to chromosome 1q31.1, a region that is often amplified in breast cancer [16, 23], we first investigated ESE-1 copy number level between normal breast tissue and breast carcinoma subtypes using DNA data available from the TCGA Breast 2 cohort (generated by the TCGA Research Network: http://cancergenome.nih.gov/) in Oncomine [24]. We found a significant increase in the log2 ESE-1 copy number units between normal breast and cancer tissues, with luminal, HER2-enriched and triple negative cancer types showing a median 1.2-1.3-fold copy number increase (Anova P value <0.001) (Figure 1A). Given the strong correlation between the level of *ESE-1* mRNA expression and copy number alterations (Supplementary Figure 1), we next determined whether *ESE-1* mRNA log2 intensity varied between breast cancer subtypes compared to normal breast tissue using the TCGA Breast dataset from Oncomine [24]. Not surprisingly, we found that the median level of *ESE-1* mRNA expression was 2.8- to 3.3-fold higher in cancer tissues compared to the normal (Anova P value <0.001) (Figure 1B). Notably Figure 1B also showed that ESE-1 expression in the HER2 subtype tumors were quite high translating to log2 values ranging from 0 to 2 at the least. Other tumor types including the HER2^+^, which consisted of both the HER2 subtype and the HER2^+^ luminal B tumors had low to high level of ESE-1 expression translating to log2 values ranging from -1 to 3.

In several breast cancer cell lines and tumor samples ESE-1 is detectable in the nucleus and/or cytoplasm [7, 8, 18, 25]. Using GOBO (Gene Ontology Based Outcome) we next analyzed for ESE-1 mRNA expression in 51 immortalized cell lines, using normalized gene expression data that have been published previously by Neve et al [26, 27]. We found that the triple negative cell lines harbored a wide range of ESE-1 expression with the log2 *ESE-1* mRNA intensity ranging from -2 to 2 (Figure 1C, left panel). Basal A and the Basal B cell lines (Figure 1C, right panel), both of which associate strongly with the triple negative subtype, reflected this diversity in ESE-1 expression. Basal A cells had high ESE-1 mRNA expression, while the Basal B cells were low or negative for ESE-1. All HER2^+^ cell lines (Figure 1C, left panel) on the other hand had a high level of ESE-1 mRNA expression translating to positive log2 mRNA expression values ranging from 0 to 1. This consistent positive expression in HER2+ cells was in contrast to the more highly variable ESE-1 expression in triple negative, hormone receptor positive cells (Figure 1C, left panel) and the luminal cells (Figure 1C, right panel); all of which showed variable ESE-1 mRNA expression levels, with log2 values ranging from -1.5 to greater than 2.5. Scoring for ESE-1 expression level in each of the individual cell lines (Supplementary Figure 2) revealed that within the luminal cell lines, the MCF7 luminal A cell line showed very low expression of ESE-1, while most HER2^+^ luminal B cells, such as BT474, ZR75-1 and ZR75-30, had higher levels of ESE-1 mRNA. The HER2 subtype AU565 and SKBR3 cells had a much higher level of ESE-1 than either of MCF7 or BT474 cells.

### High expression of ESE-1 mRNA associates with tumor subtype-specific survival outcomes

**Figure 2:**
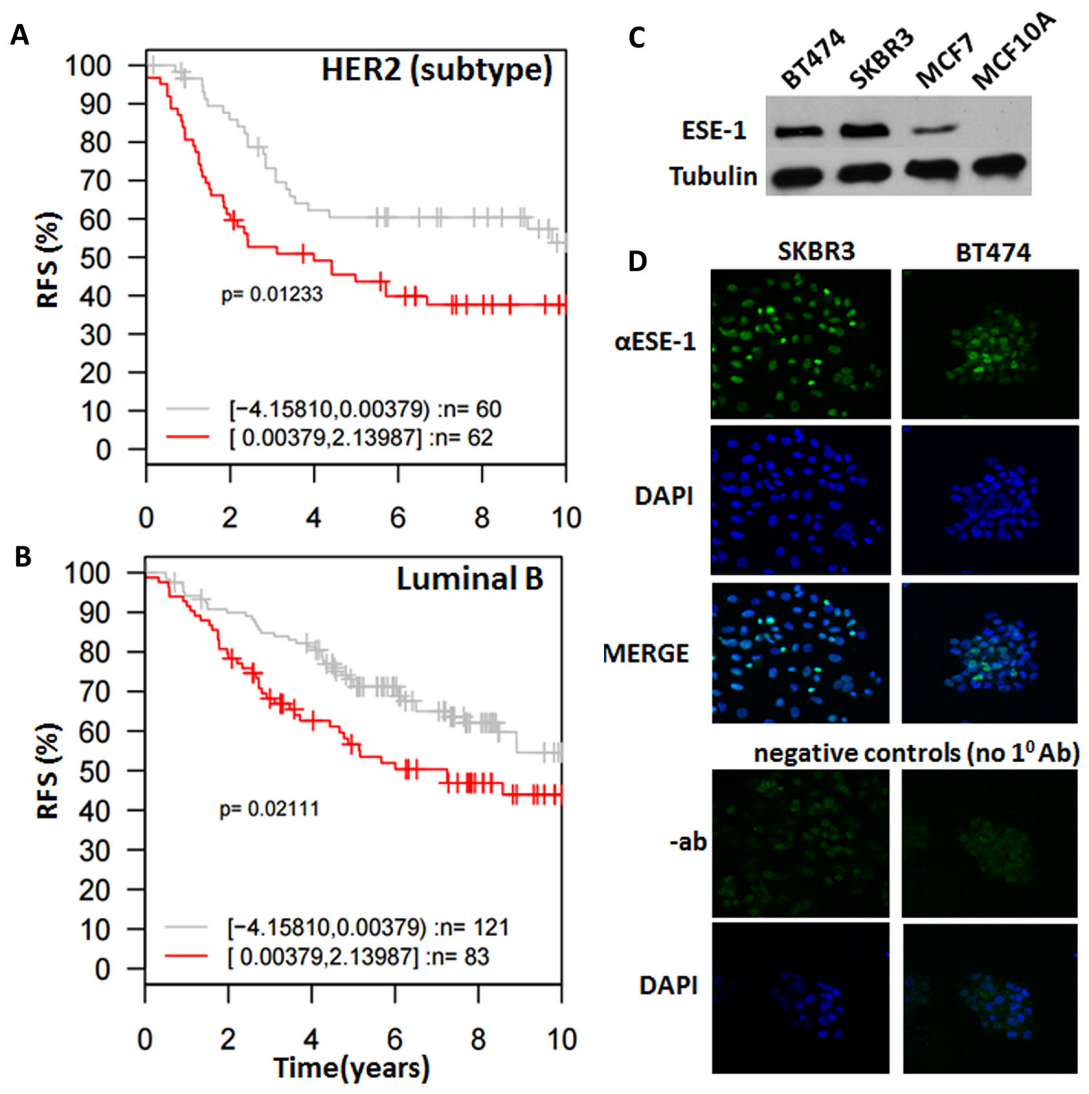
*ESE-1* dictates subtype specific clinical outcomes and is detected in the nucleus of HER2^+^ cell lines **(A, B)** Kaplan Meier survival curves of patients stratified by ESE-1 expression level and clinical subtypes of cancer generated by GOBO. PAM50 subtype classification was generated by GOBO according to gene expression subtypes reported by Parker et. al [28]. Plots show that HER2 subtype (A) and Luminal B (B) breast cancer patients that have high ESE-1 expression present experience significantly shorter relapse free survival, compared to patients with low ESE-1 expression in 10-year follow up studies. **(C)** Western blot of whole cell extracts generated from SKBR3, BT474, MCF-7, MCF10A cells and probed with the anti-ESE-1monoclonal antibody mAB405 and a mouse monoclonal anti-Tubulin. **(D)** Immuno-cytochemical analysis of ESE-1 expression in SKBR3 and BT474 HER2+ cells. The top row shows the confocal images of SKBR3 and BT474 probed with anti-ESE-1 mAb405, followed by a Cy3 conjugated goat anti-mouse secondary antibody. Both SKBR3 and BT474 are positive for green in the nucleus. Top middle row shows the same cells stained with DAPI to outline the nuclei, and the third row shows the merge of the top two rows. Shown at the bottom are the negative controls, depicting the confocal images of the same cells probed with blocking solution and the Cy3-conjugated goat anti-mouse secondary antibody but omitting the anti-ESE-1 mAb. The down bottom row shows the confocal images of DAPI stained, negative control cells.

We next determined whether ESE-1 expression is able to determine survival outcomes by Kaplan-Meier analysis using DFS (Disease free survival)/RFS (Relapse free survival) as endpoints for the different subtypes of tumors stratified into two quantiles based on *ESE-1* gene expression level using Gene ontology based outcome (GOBO) [27]. Subtype classification was generated by GOBO according to gene expression subtypes reported by Parker et. al (PAM50) [28]. We found that patients in the HER2 subtype and the Luminal B with higher ESE-1 gene expression showed significantly worse RFS than patients with low ESE-1 expression based on a 10 year follow up (Figure 2A and 2B). However, we did not find any significant association of ESE-1 gene expression with survival outcomes in the triple negative or the luminal A patients (data not shown), even though they harbor high ESE-1 mRNA level compared to normal tissues. Hazard ratio in Luminal B patients was 0.625 and hazard ratio in HER2 enriched patients was 0.6 with confidence interval <1 indicating that patients within these two subtypes with low ESE-1 had lower chance of relapsing than patients with high ESE-1 mRNA expression. Taken together, our data demonstrate the prognostic importance of ESE-1 expression level and that its utility may be closely tied to breast cancer subtypes, with its key prognostic clinical relevance being in the luminal B and the HER2 subtypes.

### ESE-1 expression is detected in the nucleus of HER2^+^ subtype SKBR3 and HER2^+^ luminal B BT474

In breast cancer tumor samples ESE-1 protein is detected both in the nucleus and cytoplasm. We therefore used our anti-ESE-1 mAb405 [8, 18] to perform Western Blot analysis and immunofluorescence cytochemistry (ICC) studies in order to confirm the endogenous expression and subcellular localization of ESE-1 in the HER2^+^ subtype SKBR3 and HER2^+^ luminal B BT474 cell lines. Western blot results revealed that both cells express ESE-1 and that ESE-1 expression is higher in these cells compared to the MCF-7 luminal A cells (Figure 2C), which was in accordance with the mRNA data and our previous reports [8]. MCF10A cells, which are non-transformed MECs, do not express ESE-1 [8, 17], and thus served as a negative control (Figure 2C). The ICC study showed that ESE-1 was detected only within the nucleus of SKBR3 and BT474 HER2^+^ cells (Figure 2D), which is consistent with what we have previously reported in MCF-7, ZR-75-1, and T47D breast cancer cell lines [8]. The lower panel of Figure 2D, which is devoid of IF signal, shows the negative control, whereby the primary α-ESE-1 mAb405 antibody was omitted.

### Knockdown of ESE-1 in SKBR3 and BT474 cells inhibits cell proliferation, 2D colony formation and anchorage independent growth

To determine the role of nuclear ESE-1 protein in controlling the transformed phenotype in SKBR3 and BT474 HER2^+^ cells, we knocked down ESE-1 using shESE-1_1.3 and shESE-1_1.5 lentiviruses, and measured cell growth, proliferation and colony forming ability. As a negative control, we used a sh-scramble lentivirus (shScr), which does not target any known transcripts of RNA. Pools of transduced cells were selected with puromycin, and then plated for each of the various assays in the absence of puromycin. We used a short selection time of 2 days to avoid counter competitive response to ESE-1 knockdown.

**Figure 3:**
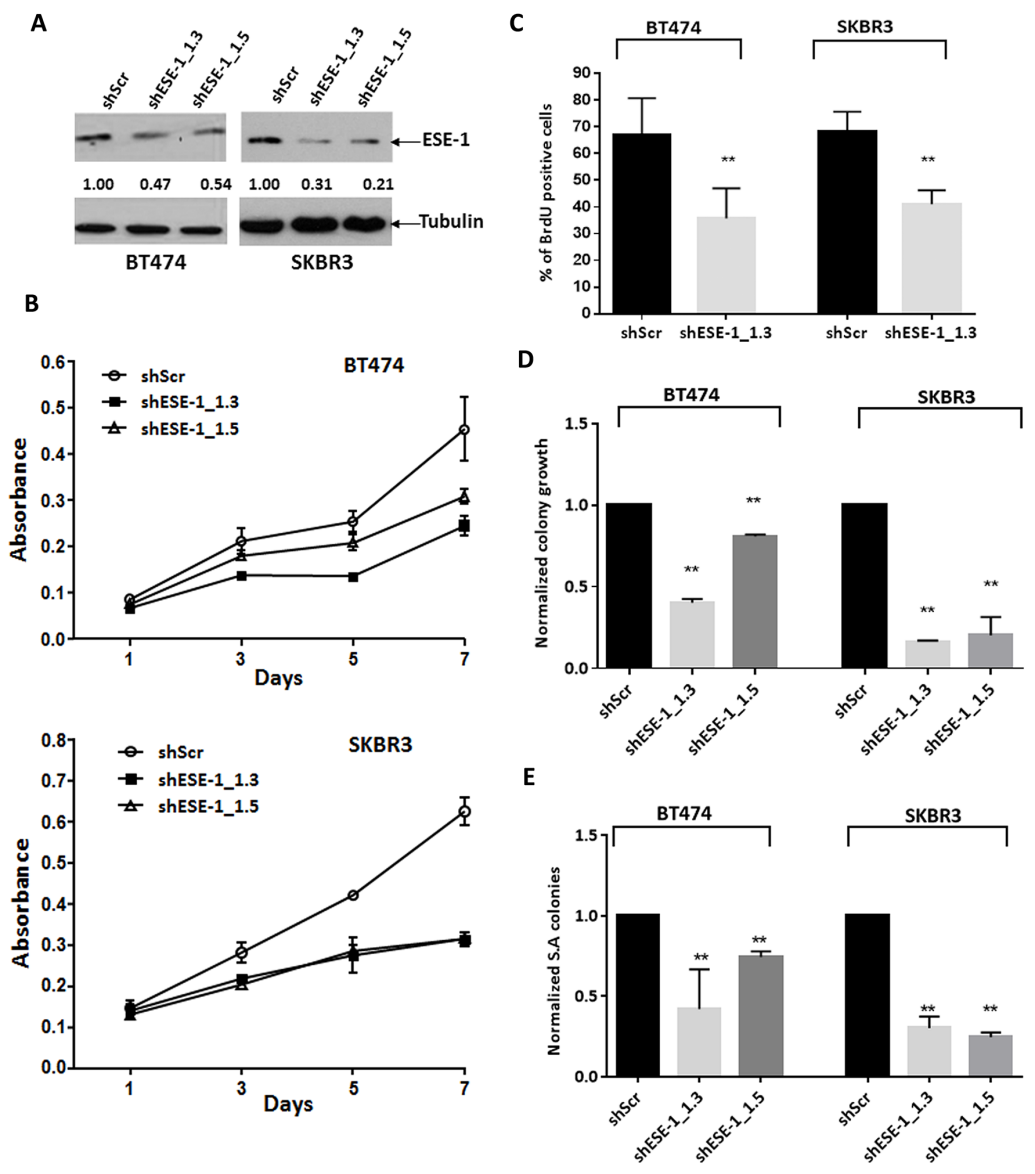
ESE-1 KD inhibits the transformation phenotype in BT474 and SKBR3 HER2^+^ cells **(A)** ESE-1 knockdown after sESE-1_1.3 and shESE-1_1.5 lentiviral transduction in BT474 and SKBR3 cells.Arbitrary densitometric units (ADU) measuring the ESE-1 densitometry signal normalized against tubulin, with the shScr set to 1, is shown under each protein signal as a number. **(B)** Cell growth assay by crystal violet staining. Data shown is a representative of four biological replicates. **(C)** Quantification of BrdU positive cells in BT474 and SKBR3 cells with stable knockdown of ESE-1, relative to the scramble control at 8 days and 3 days post transduction respectively. Figure shown is a representative of 3 replicates. Negative control was incubated with a non-specific IgG for primary antibody. The shESE-1 mediated reduction in proliferation is significant to p=0.012 in BT474 cells and to p=0.020 in SKBR3 cells over three biological replicates. **(D)** Direct quantification of BT474 and SKBR3 2D colonies stained with crystal violet using a macro built into NIH Image J software after stable knockdown of ESE-1. The shESE-1 mediated colony reduction in BT474 cells is significant to p=0.002 for shESE-1_1.3 and p=0.02 for shESE-1_1.5. The reduction is statistically significant to p=0.0002 for SKBR3 cells. Data shown are average of three independent experiments, each normalized to the scramble control, which was set to 1. **(E)** Quantification of SKBR3 and BT474 soft agar colonies stained with tetrazolium nitroblue using the Image J software. In BT474 cells, colony reduction is statistically significant to p=0.002 with shESE-1_1.3 and p=0.024 with shESE-1_1.5. The shESE-1 mediated colony reduction is statistically significant to p=0.0001 in SKBR3 cells. Data shown are an average of three independent experiments, each normalized to the scramble control set to 1. All error bars are represented as +/- SEM.

Using two distinct ESE-1 targeting shRNAs, we achieved a 53% and 46% reduction of ESE-1 in BT474, with shESE-1_1.3 and shESE-1_1.5, respectively, compared to non-targeting shRNA control (Figure 3A). Similarly, in SKBR3 cells, shESE-1_1.3 and shESE-1_1.5 expression resulted in 69% and 59% ESE-1 protein knockdown, respectively (Figure 3A). In both cell types, shESE-1_1.3 elicited a better knockdown. Cell growth assays showed an equivalent and average growth reduction of 46% by shESE-1_1.3 in the BT474 and about 50% by both shESE-1_1.3 and shESE-1_1.5 constructs in SKBR3 cells by day 7 (Figure 3B). In contrast, the decrease in growth rate in the BT474 by shESE-1_1.5 was less, with 33% decrease by day 7 (Figure 3B). BrdU was next used as a direct marker to assess cellular proliferation (Figure 3C). ICC staining at day 8 revealed a 32% decrease (p = 0.012) in the ESE-1 KD BT474 cells and a 27.5% reduction (p = 0.02) in the number of BrdU^+^ cells in the ESE-1 KD SKBR3 cells compared to the control at day 3 (Figure 3C).

We next performed clonogenicity studies, plating an equal number of live shScr and shESE-1 cells at low density in complete media. ESE-1 KD in BT474 cells resulted in 60% reduction (p=0.002) in clonogenicity with shESE-1_1.3 and a 20% decrease (p=0.02) with shESE-1_1.5 (Figure 3D). Knockdown in SKBR3 cells resulted in an 80% reduction (p=0.0002) in colony numbers for both shESE-1 constructs (Figure 3D). To assess the effects of ESE-1 KD on transformation properties, we used anchorage independent growth in soft agar. As in the clonogenicity studies, ESE-1 KD in BT474 cells resulted in 53% reduction (p=0.002) in soft agar colonies with shESE-1_1.3 and a 20% decrease (p=0.024) with shESE-1_1.5 (Figure 3E). To assess whether the blunted responses noted in BT474 cells could be due to a threshold effect, and that a more complete ESE-1 knockdown would elicit a more robust response, we used siRNAs to knock down ESE-1. These siRNA studies showed a more complete ESE-1 KD, and a more efficient reduction in cell proliferation and soft agar colony formation in BT474 cells (Supplementary Figure 4). Knockdown in SKBR3 cells resulted in a 75% reduction (p=0.0001) in soft agar colony number in SKBR3 cells with each shESE-1 construct (Figure 3E).

### ESE-1 knockdown does not induce apoptosis but causes a cell accrual in the G1 phase

**Figure 4:**
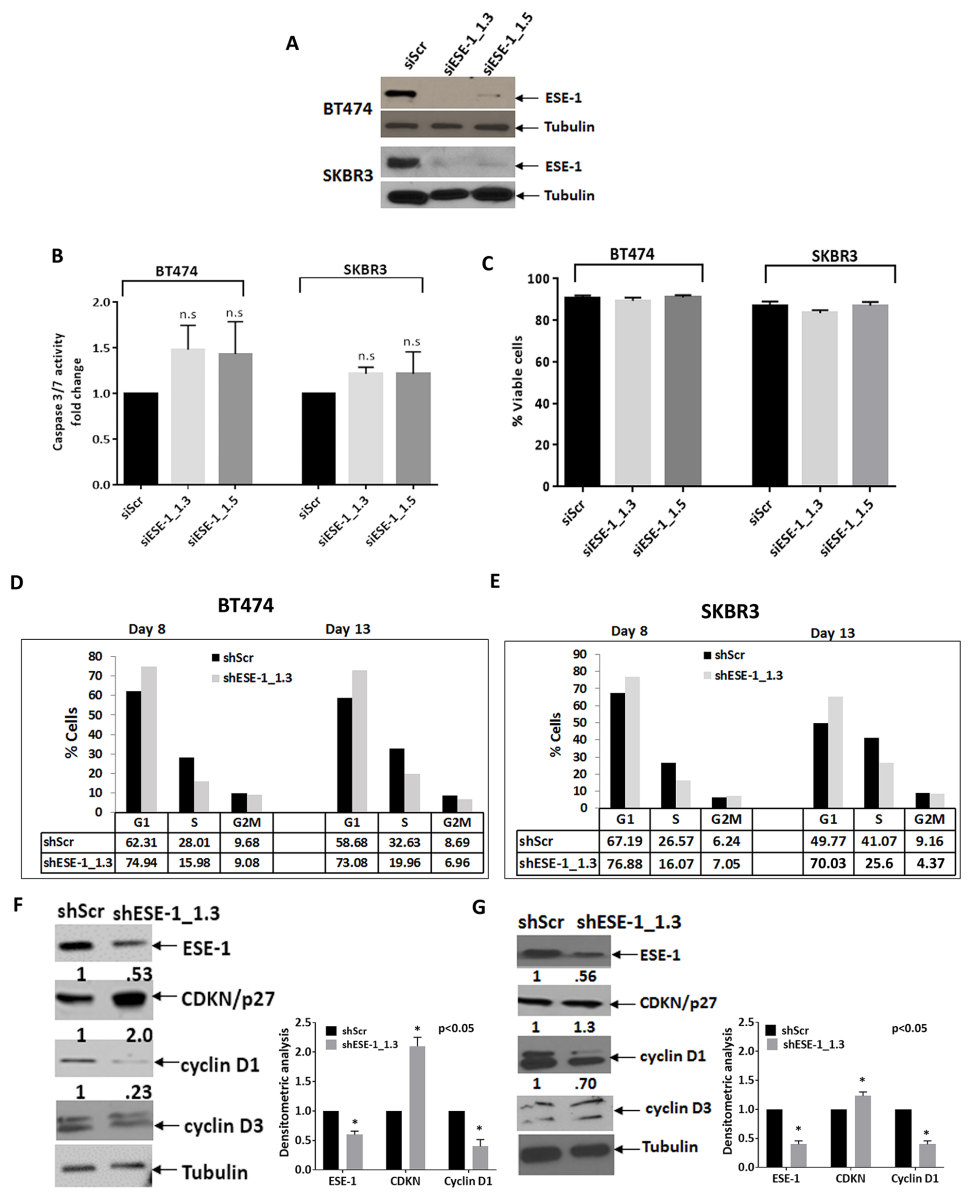
ESE-1 KD does not affect apoptosis but causes a delay in progression through G1 **(A)** A Western blot showing transient ESE-1 knockdown using siESE-1_1.3 and siESE-1_1.5 in BT474 and SKBR3 cells, 3 days post transfection. Transfection with siESE-1 (70 nM) almost completely inhibits ESE-1 expression. **(B)** Caspase 3/7 activity at day 3 post-transfection in BT474 and SKBR3 cells. The caspase 3/7 activity of the scramble control was set to 1, and that of the siESE-1 transfected cells were normalized to the scramble control value and expressed as fold change. There is no statistically significant difference between the knockdown and the control. In BT474 cells, the p values for the si-ESE-1_1.3=0.080 and for siESE-1_1.5=0.170 and in SKBR3 cells the p values for the si-ESE-1_1.3=0.148 and 0.427 for siESE-1_1.5. **(C)** Viability of the siESE-1 transfected BT474 and SKBR3 cells. Viability was measured by the trypan blue exclusion method using the Beckman coulter Vi-cell. **(D)** Cell cycle analyses of the DNA content were done on a synchronized cell population of BT474 cells. **(E)** Cell cycle analyses in SKBR3 cells were done on an unsynchronized cell population. Cell cycle analysis was performed, as detailed in Methods. The numbers on the Y axis represent the percent of cells at G1, S, and G2M for the knockdown and the scramble control, and are representative of three or more biological repeats. The cells were collected at 8 days and 13 days post transduction. The p-values were calculated using all three replicates (see Supplementary Figure and text). ESE-1 KD cells progress through the G1 slowly compared to the scramble controls. All p values were estimated from the results of 3 experiments. **(E, F).** Western blot with anti-cyclin D1, anti-cyclin D3, anti-p27^kip1^ on a portion of the cells collected at day 8.

To begin to address the mechanism by which knocking down ESE-1 exerts its growth inhibitory effects, we performed apoptosis assays. We chose the siRNA approach, using siESE-1_1.3 and siESE-1_1.5, to achieve more complete knock down of ESE-1. Furthermore, these apoptosis assays are short term and do not require stable ESE-1 knockdown. Western blot analysis confirmed that the siRNAs nearly completely eliminated ESE-1 expression in both cell lines at day 3 post-transfection (Figure 4A). The caspase 3/7 apoptosis assay results was normalized over 3 biological replicates. Although there is a slight trend to activation of caspase 3/7 activity upon knock down of ESE-1 in both cell lines, the results were not statistically significant compared to the siScr control, indicating that ESE-1 KD does not induce apoptosis (Figure 4B). This was also consistent with our previous work showing that ESE-1 KD does not elicit an apoptotic response [8]. The percentage of viable cells was also not significantly different between the ESE-1 knockdowns and the siScr 72 hours post-transfection, as depicted in Figure 4C, further ruling out apoptosis as a response to ESE-1 knockdown.

We next conducted cell cycle assays in both cell types post-infection days 8 and 13. Figure 4D and 4E show representative data from single cell cycle analysis study in BT474 and SKBR3 cells, respectively. BT474 cells continued to grow in low serum media, failing to fully synchronize at G0. Thus, we used a lovastatin-mevalonate rescue approach in BT474 cells [29]. Figure 4D shows a ∼12% G1 accumulation at day 8 and a ∼14% G1 accumulation at day 13. Including multiple biological replicates shows that ESE-1 KD in synchronized BT474 cells accumulated cells in the G1 phase with an accrual of 114% and 112% at days 8 and 13, respectively, compared to shScr set at 100% (Supplementary Figure 5B). Figure 4E shows that ESE-1 knockdown in unsynchronized SKBR3 cells at day 8 increased the percent of cells halted at G1 (76.9%), compared to the scramble control (67.2%), resulting in an accrual of ∼9%, and at day 13, shESE-1 KD cells at G1 was 70.03% compared to 49.8% for the shScr, for an accrual of ∼20%. Supplementary Figure 5A is the flow cytometry data for that study. On completing three or more biological replicates of these cell cycle studies, we found an average accrual of 113% at day 8 with ESE-1 KD compared to shScr set at 100%, and an average accrual of 128% at day 13 (Supplementary Figure 5B).

Since ESE-1 KD resulted in G1 accumulation, we next assessed whether ESE-1 KD altered the levels of checkpoint proteins important for the G1 to S phase transit analyzed in cell extracts collected at day 8 post transduction. ESE-1 KD inhibited cyclin D1 and showed a definite accumulation of the CDKI p27^Kip1^ (Figure 4F and 4G) in both cell types, indicating a common role of cell cycle checkpoint proteins in ESE-1mediated transformation. Analysis of whole cell extracts at a similar time point showed that knocking down ESE-1 had no effect on cyclin D3 in either cell line (Figure 4F and 4G).

### Stable ESE-1 knockdown inhibits active Akt to inhibit proliferation

Previous reports have shown that ESE-1 regulates HER2 expression and that small molecule interference of the ESE-1/Sur2 interaction inhibits HER2-mediated downstream signaling [20–22]. Thus, we tested if the key mechanism by which ESE-1 mediates transformation is through regulation of HER2 protein expression and HER2 downstream signaling molecules. Transient inhibition of ESE-1 using siESE-1_1.3 down-regulated HER2 protein expression in BT474 and SKBR3 cells as early as 48-hours and 72-hours post-transfection, respectively (Supplementary Figure 6). However, reduction of HER2 protein did not block the Tyr1221/1222 phosphorylation of HER2 or affect downstream Akt activation in BT474 cells. In contrast, inhibition of HER2 in SKBR3 cells 72-hours post-transduction resulted in downregulation of active HER2, active HER3, Akt and pAkt (Serine 473). Transient ESE-1 down-regulation did not affect active ERK in either of the cell lines (Supplementary Figure 6).

**Figure 5:**
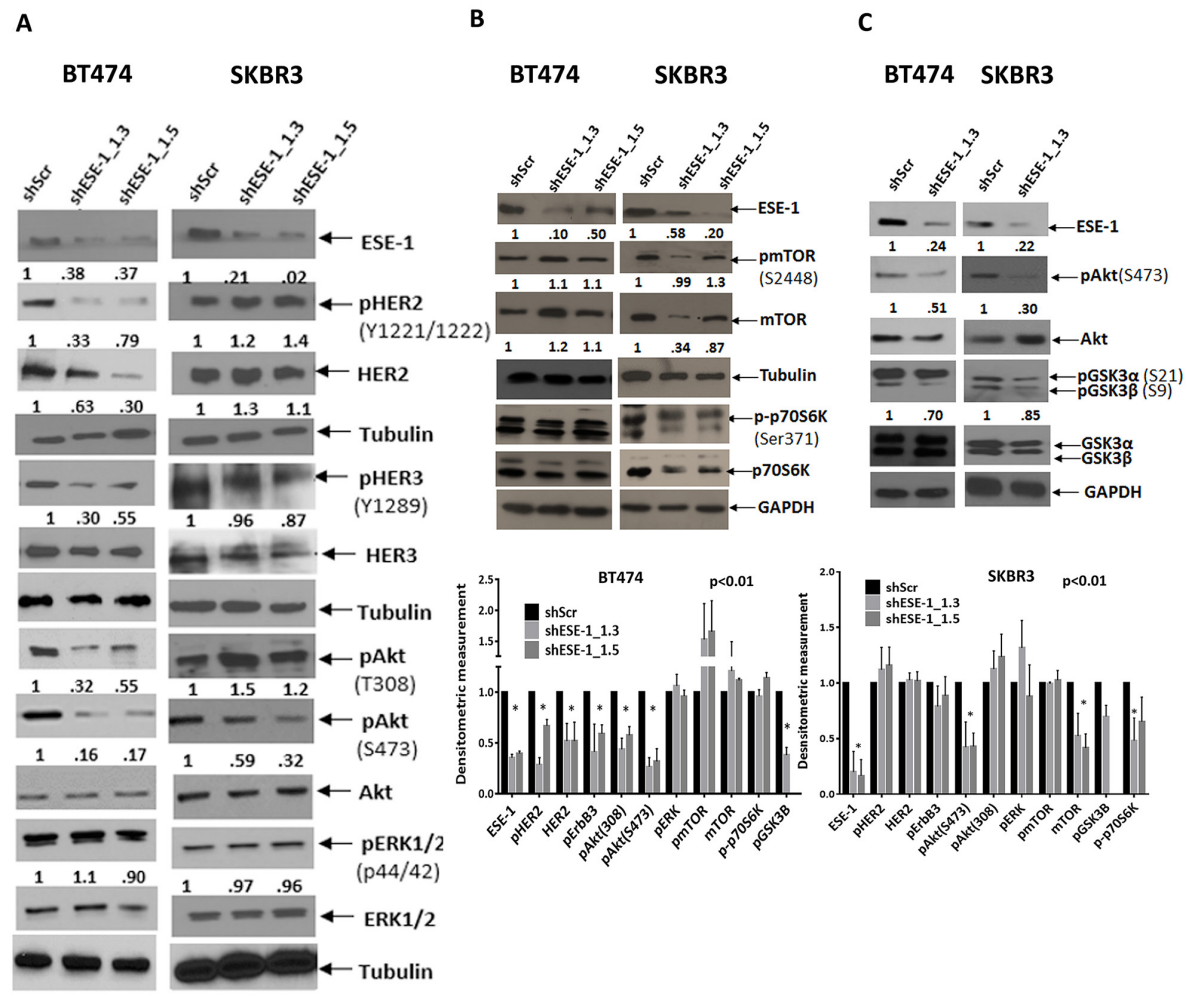
Persistent ESE-1 KD differentially affects signaling in BT474 and SKBR3 cells **(A)** BT474 and SKBR3 cells were stably transduced with shESE-1_1.3 and shESE-1-1.5, and cell extracts were immunoblotted with anti-ESE-1, anti-pHER2 (Tyr1221/1222), anti-total HER2, anti-pHER3 (Y1289), anti-HER3, anti-pAKT (Thr 308 and Ser473), anti-total AKT, anti-pERK1/2(p44/42), anti-total ERK, and anti-Tubulin at 8 days post transduction. **(B, C)** Whole cell extracts collected at day 8 post transduction were blotted for anti-pmTOR(S2448), anti-mTOR, anti-p70pS6K, anti-pAkt, Akt, anti-pGSK3α,β (S21 and S9) and anti-tubulin. Quantitations of all nonphosphorylated protein forms were done by normalizing to tubulin or GAPDH. Phosphorylated protein forms were normalized to tubulin or GAPDH prior to normalization to the normalized total of the same. Extra tubulin or GAPDH controls have been provided at the bottom of few blot segments to note any loading inconsistencies that have occurred in between gels.

We next looked at stable ESE-1 downregulation to understand the mechanism underlying the long term anti-proliferative effect of ESE-1 knockdown. Stable transduction of BT474 and SKBR3 cells with shESE-1_1.3 and shESE-1_1.5 constructs resulted in ≥ 50% knockdown of ESE-1 (Figure 5A). Stably knocking down ESE-1 at day 8 post-transduction in BT474 decreased HER2 protein expression, HER2 auto-phosphorylation (Tyr1221/1222), HER3 transphosphorylation possibly leading to a decrease in the level of active Akt (pThr308 and pSer473) (Figure 5A). In contrast, stable knockdown of ESE-1 in SKBR3 cells resulted in minimally induced HER2 and pHER2 expression when compared to the scramble control (Figure 5A), although quantitation of pHER3 still showed an 8% average decrease in phosphorylation with both shESE-1’s. Since only HER3 phospho-tyrosine sites can directly bind the p85 unit of PI3K [30], we tested if this small decrease in HER3 phosphorylation inhibits the PI3K-PDK1 mediated phosphorylation of Akt at Thr308. Instead, we found an increase in phosphorylation at Thr308, indicating that either HER3 transphosphorylation was not being adequately inhibited to block phosphorylation at Thr308 or the increase in pThr308 was a response to adaptive signaling. There was, however, a 44% decrease in Akt phosphorylation at Ser473, which was consistent across several biological replicates. Phosphorylation of ERK1/2 (p44/42) in both cell types showed little to no change, suggesting that the long-term anti-proliferative effect of ESE-1 knockdown is primarily a result of inhibition of active Akt.

Since mTORC2 phosphorylates Akt at Serine 473, we next tested if downregulation of ESE-1 influences mTOR protein levels or mTOR activation. While BT474 cells evinced little or no change in mTOR protein level when normalized to tubulin, there was a consistent decrease in mTOR protein expression in SKBR3 cells upon ESE-1 knockdown across replicates (Figure 5B). The level of pmTOR, when normalized to mTOR protein level, showed no quantitative change, indicating that the inhibition of pmTOR is a result of decreased mTOR protein expression. Because mTOR protein is a part of the mTORC1 and mTORC2 complexes, we investigated whether mTOR down-regulation inhibited Ser371 phosphorylation of p70S6kinase protein, a substrate of mTORC1. In BT474 cells, we observed little or no decrease in phosphorylation of Ser371 in the knockdown compared to the scramble control. In SKBR3 cell lines there was a modest inhibition of phosphorylation at Ser371 in SKBR3 in the two shESE-1’s, establishing that inhibition of mTOR protein adversely affects the kinase activity of the mTORC1 complex.

In spite of the differential signaling, long term inhibition of ESE-1 attenuated the phosphorylation of Akt at both Thr308 and Ser473 in BT474 cells, and partially blocked Akt activation by inhibiting Ser473 phosphorylation in SKBR3 cells. This prompted us to examine if downstream Akt signaling was affected in the two cell types. In both cell lines, inhibition of pAkt paralleled with inhibition of total pGSK3α and β (Figure 5C). Specifically, BT474 cells showed a total decrease of 30% in GSK3 (α+β) phosphorylation compared to the scramble control, with phosphorylation of GSK3β being largely diminished compared to that of GSK3α. Phosphorylation levels in SKBR3 cells were equally inhibited in both isoforms resulting in a total decrease of 15% (pGSK3 α and β) in comparison to the scramble control.

### Constitutively active Akt rescues colony formation in ESE-1 knockdown BT474 and SKBR3 cells

**Figure 6:**
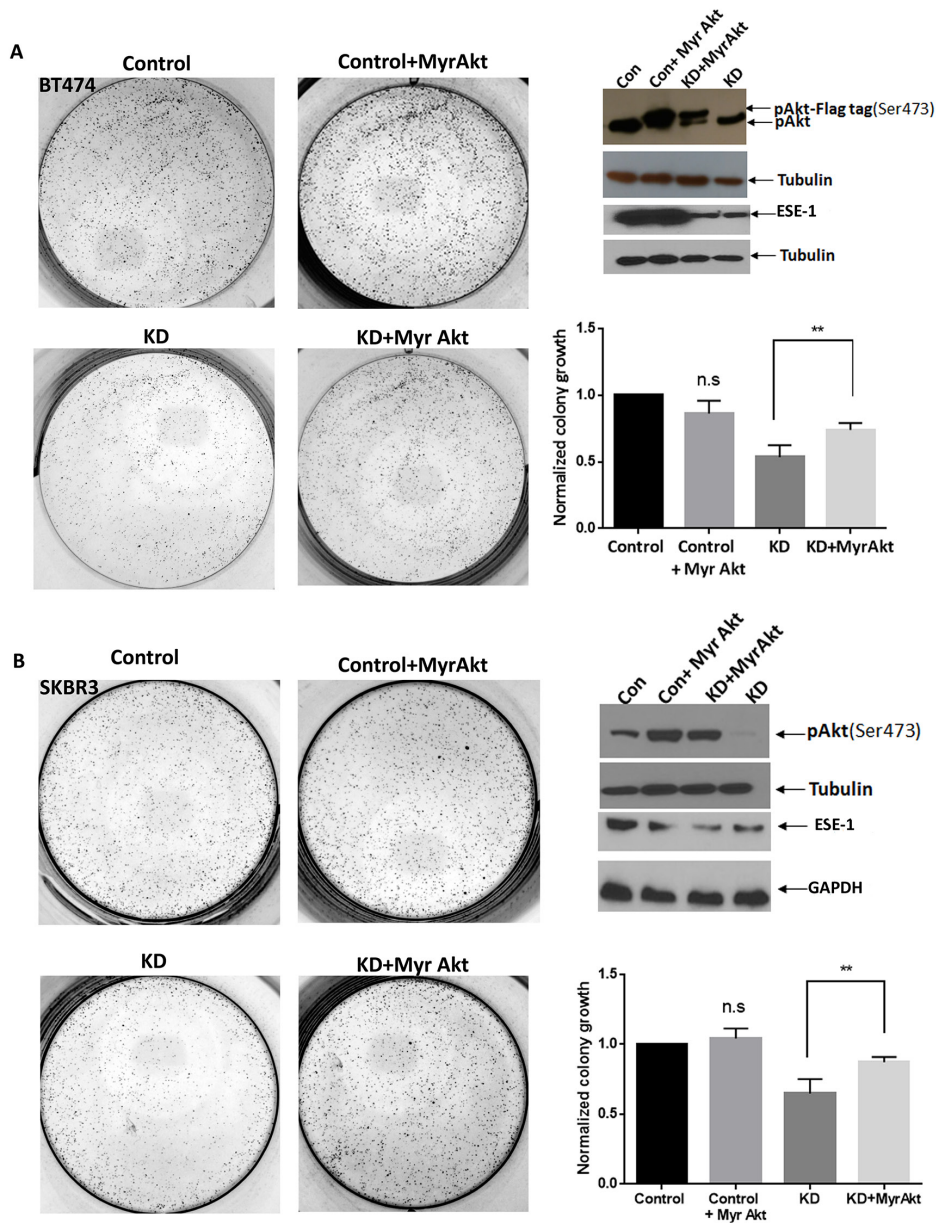
Constitutively active Akt partially rescues ESE-1 KD mediated inhibition in growth **(A)** BT474 cells were transfected with a Flag tagged Myr-AKT expressing plasmid or the vector control followed by transduction with the scramble control or shESE-1_1.3. **(B)** SKBR3 cells were transfected with a Myr Akt (untagged) plasmid or the vector control followed by transduction with the scramble control or shESE-1_1.3. In both graphs, cells labeled as Control bears the scramble control, cells labeled as Control + Myr Akt bears the scramble control and the Myr Akt plasmid. Cells labeled KD + Myr Akt bears shESE-1_1.3 and Myr Akt. Data shown are the average of three independent experiments, each of the Con + Myr Akt, and the KD, was normalized to Control. The KD+Myr Akt was normalized to Control+Myr Akt. The value of Control was set to 1. The p-value (p=0.027) for BT474 cells and p=0.022 in SKBR3 cells were determined using three replicates for each cell type by an unpaired T test in Graphpad prism. Error bars are +/- SEM derived from all three experiments.

To directly test the role of active Akt in ESE-1-mediated proliferation, we next examined if constitutively-active Akt rescues the anti-proliferative effect of ESE-1 KD in BT474 and SKBR3 cells (Figure 6). We transfected BT474 and SKBR3 cells with Myr-Akt-Flag and Myr-Akt, respectively, followed by transduction with shESE_1.3 or the scramble control. Cells were maintained under puromycin and G418 treatment to select for cells expressing both Myr-Akt and shESE_1.3 or Myr-Akt and scramble control, and cell lysates were collected 8 days post-transduction. In both cell types, transduction of shESE-1_1.3 inhibited pAkt, as indicated by a reduction of pSer473Akt relative to controls (Figure 6A and 6B). However, in control cells transfected with Myr-Akt-Flag (BT474 cells) or Myr-Akt (SKBR3 cells) resulted in increased pAkt levels compared to control cells. Transfection of ESE-1 KD cells with Myr-Akt-Flag (BT474 cells) or Myr-Akt (SKBR3 cells) resulted in a rescue of pAkt levels. Furthermore, in 2D clonogenic assays completed over 11 days, transfection with Myr-Akt-Flag (BT474 cells) or Myr-Akt (SKBR3 cells) in ESE-1 knockdown cells partially rescued the anti-proliferative effect of ESE-1 KD in BT474 and SKBR3 cells (Figure 6A and 6B), further validating that Akt is a downstream target of ESE-1 and that the anti-proliferative effect of ESE-1 KD is in part mediated through regulation of pAkt.

### Knocking down ESE-1 inhibits HER2^+^ xenografts in NOD/SCID mice

**Figure 7:**
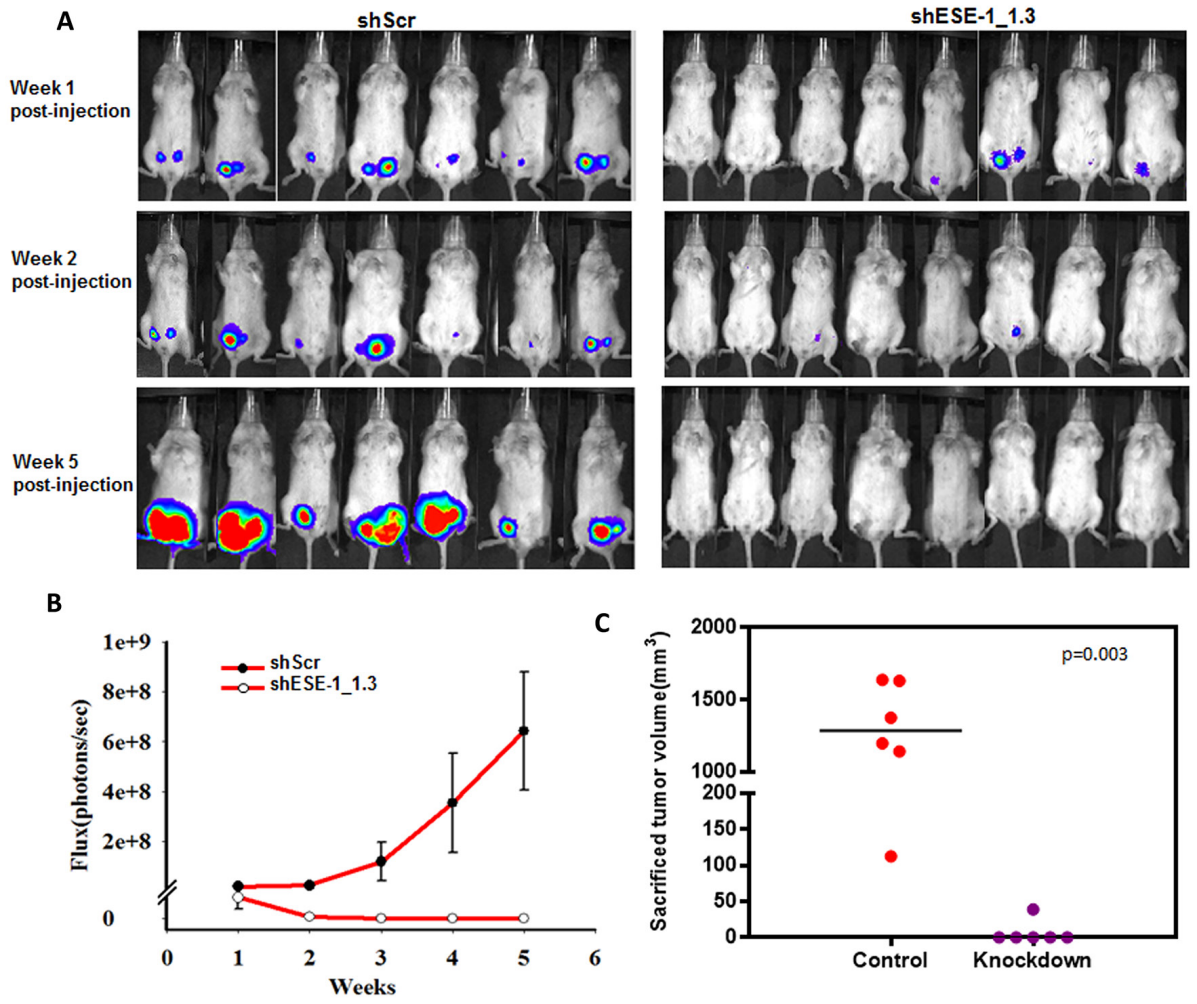
ESE-1 KD inhibits tumor formation in NOD/SCID female mice **(A)** BT474 cells transduced with either shScr or shESE-1 were injected into mammary fat pads of NOD/SCID mice to establish tumor xenografts. Tumor growth is shown as IVIS signaling at weeks 1, 2 and 5 post injection. **(B)** Total photon flux from tumors derived from shScr control cells and derived from shESE-1 knocked down cells is shown over 5 weeks. **(C)** Sacrificed tumor sizes in shSchr control and shESE-1 knockdown groups. Tumors sizes were measured using the modified ellipsoid formula, Tumor volume = ½(length * width^2).

Given the effects of ESE-1 KD on reducing the transformed phenotype in two HER2^+^ breast cancer cell lines, we next sought to determine whether ESE-1 knockdown could inhibit xenograft tumor formation of BT474 cells in NOD.SCID mice. We focused on BT474 cells, since SKBR3 cells do not form tumors. Also, the BT474 cells are luminal ER+, HER2 subtype, and we implanted the recipient mice with estrogen pellets to further optimize tumor growth [31]. In the 7 mice injected into the left and right #4 mammary fat pads with BT474 cells stably transduced with the shScr control, we detected 12 of 14 tumors via luciferase flux at week 1 (Figure 7A, top left panel). In contrast, injection of the shESE-1_1.3 KD cells into 8 mice into the left and right #4 mammary fat pads resulted in only 4 of 16 potential tumors at week 1 (Figure 7A, top right panel). By week 2, we detected by luciferase flux 10 nonpalpable tumors in the shScr control mice, and these 10 continued to grow by week 5 (Figure 7A, top left panel). In contrast, by week 2, only one of the original four signals was weakly retained in the mice injected with ESE-1 KD cells, and this completely vanished by week 5, such that none of the mice had any detectable flux (Figure 7A, top right panel). Furthermore, we measured the total flux of all tumors on a weekly basis in the shScr control and shESE-1_1.3 KD mice, showing the rapid growth rate of the shScr BT474 tumors compared to the complete lack of growth ESE-1 KD BT474 tumors (Figure 7B). At 5 weeks the total flux for shScr tumors was 6.5e+8 and for the ESE-1 KD tumors was 0 (Figure 7B). These results show that knocking down ESE-1 inhibited tumor formation in all 16 potential tumor sites, as measured by IVIS. Mice were sacrificed at week 8 and excised tumor volumes were measured using calipers. Three mice in the control group, harboring 4 tumors, died at weeks 6 and 7, and these tumors were not included in the size measurement. The average tumor size in the control group was 1000 mm^3^ (Figure 7C). Whereas, we found a single 25 mm^3^ growth that was not detectable by photon flux in the ESE-1 KD mice, and we could not measure any tumors in the remaining mice (Figure 7C).

### Knocking down ESE-1 inhibits tumorsphere formation in HER2^+^ cells

**Figure 8:**
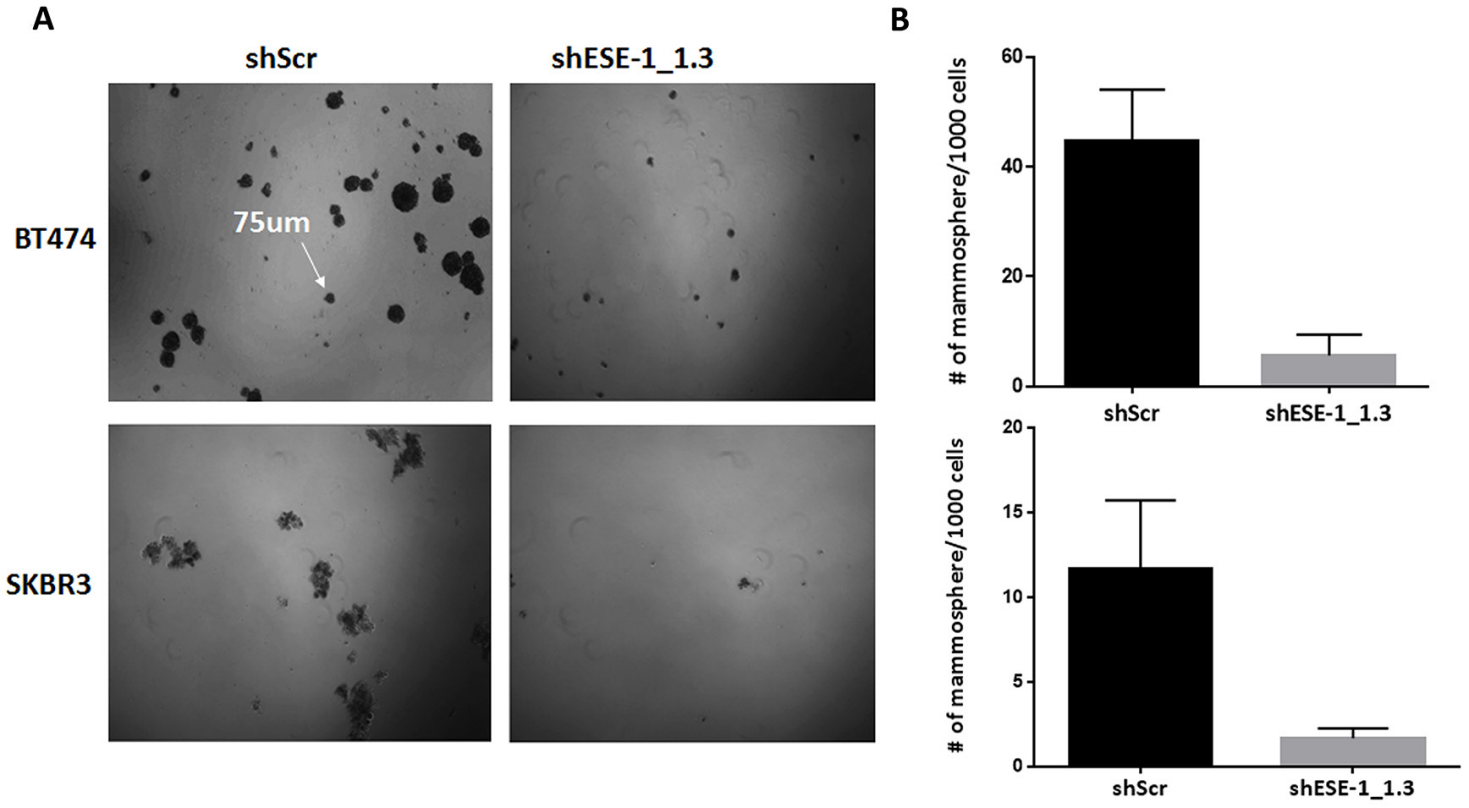
ESE-1 KD inhibits tumorsphere formation in BT474 and SKBR3 cells. **(A)** Representative field of second passage tumorspheres in the scramble control and ESE-1 KD cells. **(B)** Tumorsphere greater than 75um was counted.There is 86% decrease in the number of tumorspheres in BT474 cells and 88% reduction in SKBR3 cells when ESE-1 is knocked down. Error bars are +/- SEM derived from three biological repeats.

Given the complete inhibition of tumor formation in NOD/SCID mice upon ESE-1 knockdown, we next investigated if ESE-1 has a role in tumor initiation. We used the tumorsphere formation assay as a surrogate to assess the ability of ESE-1 knockdown cells to serve as tumor initiating cells [32]. Stable knockdown of ESE-1 inhibited tumorsphere formation in SKBR3 and BT474 cells *in vitro* by ∼86%. Figure 8A shows representative fields of tumorspheres for the scramble control and the ESE-1 knockdown cells in BT474 and SKBR3 cell lines. Quantitation of tumorspheres in three biological replicates revealed that knocking down ESE-1 led to an 88% decrease in second generation tumorsphere formation in BT474 cells (Figure 8B, top panel) and an 86% decrease in SKBR3 cells (Figure 8B, bottom panel). The near complete inhibition in tumorsphere formation suggests that ESE-1, like other ETS factors, has a role in controlling ability of cells to initiate and maintain tumors and that knockdown of ESE-1 possibly inhibits the proliferation of tumor initiating cells in HER2^+^ cells similar to anti-cancer drugs, such as Lapatinib or Metformin [33, 34] along with other . QPCR validation of gene expression analysis in control and knockdown cell suggest that ESE-1 controls expression of genes other than HER2 in the two cell lines (Supplementary Figure 7), which in turn could contribute to the growth inhibitory phenotype observed in tumor xenografts.

## DISCUSSION

Members of ETS transcription factor family have long been implicated in tumorigenesis. In humans, ETS factor fusions such as *EWS-ETS*, *ETS-Abl*, *TMPRSS6-ERG* are associated with malignancies in soft tissue, blood, and prostate, respectively [4]. Surprisingly, however, other than ETS1 and ESE-2 there is little support for a direct role of other ETS proteins in human mammary tumorigenesis [35–39]. Over the years, using benign and transformed MECs, we have established a pro-tumorigenic role for ESE-1 in breast cancer using *in vitro* approaches [7, 8, 18, 40]. However, the relevance of ESE-1 in clinical outcomes and the role of ESE-1 in *in vivo* breast cancer xenograft tumor formation is yet to be determined. In this paper, we established the clinical significance of ESE-1 expression in specific breast cancer subtypes and elucidated a mechanistic role for ESE-1 in these subtypes using a preclinical cell culture model and *in vivo* xenograft tumor formation.

The ESE-1 mRNA is over-expressed in a majority of breast carcinomas (Supplementary Figure 3), including all breast cancer subtypes. Through data mining we found that even though triple-negative and luminal breast tumors and cell lines harbor a wide range of ESE-1 mRNA expression, starting from very low or negative log values to very high, and with only HER2^+^ breast tumors and cell lines segregating with very high mRNA expression of ESE-1 (Supplementary Figures 1–3). GOBO analysis revealed that the high expression of ESE-1 in HER2^+^ cells has clinical significance (Figure 2). Patients expressing high ESE-1 mRNA have a significantly poorer RFS than their ESE-1-negative counterparts and, up to now, the data suggest that this association is most important for the HER2 subtype and the luminal B tumors, which are often HER2^+^, suggesting that the high expression of ESE-1 in a HER2-driven environment predicts a worse clinical outcome. It is yet to be addressed how ESE-1 protein expression controls survival in breast cancer patients. Ongoing work in our laboratory suggest that ESE-1 is expressed in the cytoplasm and nucleus in patient tumor samples although we have not been able to detect ESE-1 in the cytoplasm of transformed cell lines that we have tested. Previous work published by our lab has shown that ESE-1 initiates transformation in non-transformed mammary epithelial cells via a cytoplasmic mechanism but in transformed cell lines it localizes to the nucleus. The presence of cytoplasmic ESE-1 in clinical samples could therefore be linked to early events of tumor initiation and we surmise that as transformation progresses, ESE-1 is expressed in the nucleus. As mentioned, further work elucidating the correlation of subcellular ESE-1 protein expression and clinical outcomes in distinct breast cancer subtype specimens is currently being pursued in our laboratory.

Given the association of ESE-1 mRNA expression and outcomes in HER2+ tumors, in this paper, we focused our studies on two different subtype specific HER2^+^ cell lines, the ER^+^ luminal B HER2^+^ BT474 and the ER^-^HER2 subtype SKBR3 cells and demonstrated that ESE-1 controls the transformed phenotype, albeit via distinct mechanisms. Since HER2 signaling induces ESE-1 gene expression, and ESE-1 protein functions as a trans-activator of the HER2 promoter, it has been reported that HER2 and ESE-1 operate in a feed-forward mechanism [20, 21]. It has also been shown that the anti-tumor effect of targeting the ESE-1/Sur2 interface is mediated through downregulation of HER2 and HER2 signaling in SKBR3 cells harvested 72 hours from the time of ESE-1/Sur2 disruption [22]. Here we have shown through transient and stable knockdown of ESE-1 protein that although the ESE-1/HER2 relationship exists, it is more complicated than previously appreciated and manifests distinct temporal responsiveness in a cell type-specific manner in the luminal B BT474 and the HER2 subtype SKBR3 cells. These data indicate that these two cell types, while similar with regards to HER2 positivity, possess uniquely responsive signaling pathways and ER requirements to maintain the transformed phenotype; nonetheless, ESE-1 appears to control certain key nodes, such as Akt and cyclin D1, which are common to both cell types and required for transformation.

In BT474 cells, our data indicate that persistent downregulation of ESE-1 suppresses the transformed phenotype dominantly via inhibition of HER2 and effectors of HER2 signaling, such as pHER2, pHER3, pAktThr 308 and pAktSer473. Although transient knockdown of ESE-1 with siRNA down-regulates HER2 expression, only a minimal inhibition of pHER2 occurs at 72-hours post-transfection. In SKBR3 cells, persistent inhibition of ESE-1 leads to inhibition of pAKT-S473 and an induction of HER2 protein expression, whereas transient siRNA mediated inhibition of ESE-1 results in a pronounced inhibition of pHER2, pHER3 and pAkt at 72-hours post-transfection. Irrespective of the differential effects on HER2 protein expression, persistent ESE-1 knockdown consistently inhibits phosphorylation of Ser473 on Akt in both cell lines, revealing that ESE-1 knockdown affects a key pro-tumorigenic signaling node like Akt. In support of the notion that Akt is key to both cell types, we show that a constitutively active Myr-Akt partially rescues the transformed phenotype in both cell types. While this shows that ESE-1 presumably works upstream of Akt kinase and that Akt is clearly important, nevertheless, Akt may be only one of several ESE-1-regulated pathways leading to transformation.

The mechanism underlying inhibition of Akt phosphorylation at both Thr308 and Ser473 in BT474 cells is most likely via inhibition of HER2, pHER2, and pHER3. The inhibition of pAkt leads to inhibition in phosphorylation of its downstream substrate GSK3β, which is a known player in tumorigenesis. In contrast, in SKBR3 cells, the mechanism by which persistent knockdown of ESE-1 leads to inhibition of pAkt-Ser473 is complex and is plausibly mediated via reduction of mTOR. mTOR is a component of both mTORC1 and mTORC2, and it has been established mTORC2-dependent phosphorylation of Akt on Ser473 is specifically important for Heregulin mediated transformation in SKBR3 cells [41, 42]. Our data reveals that in SKBR3 cells ESE-1 knockdown inhibits mTOR protein expression and decreases p-mTOR, which then likely impairs the activity of both mTORC1 and mTORC2 complexes. Because the Akt phosphorylation site on Ser473 is a bona fide substrate of mTORC2, inhibiting the mTORC2 complex activity leads to inhibition of Ser473 phosphorylation on Akt. We also demonstrate that inhibition of ESE-1 inhibits phosphorylation of p70S6 kinase at Ser371, a site that has been shown to be phosphorylated by mTORC1 and GSK3 [43], indicating that mTORC1 activity is also attenuated upon ESE-1 knockdown in SKBR3 cells. Once mTORC1 and mTORC2 activities are inhibited, then any active HER2 signaling is uncoupled from further Akt activation, which explains the inhibition of the transformed phenotype upon ESE-1 knockdown in SKBR3 cells, even in the face of HER2 and pThr308 induction. Although we are unable to explain what specifically induces HER2 upon stable knockdown of ESE-1 in the SKBR3 cells, compensatory mechanisms arising from disruption of mTOR certainly seem possible. There is evidence that inhibition of mTORC1 with Rapamycin in pancreatic cancer cells induces HER2 expression and HER2 phosphorylation [44]. Notably, we do not observe a similar decrease in the level of mTOR protein or mTOR activation in BT474 cells, suggesting that mTOR is likely to be regulated indirectly by ESE-1 in the HER2 subtype SKBR3 cell lines.

Other key findings common between the two cell lines are that ESE-1 knockdown diminishes cell cycle progression and alters CDKI p27Kip1 and cyclin D1 checkpoint protein expression. In both BT474 cells and SKBR3, ESE-1 knockdown caused an increase in the level of CDKI p27^Kip1^, along with a decrease in the level of cyclin D1 (Figure 4E and 4F). Studies with other ETS proteins, such as ETS-2, have shown that a similar down-regulation of cyclin D1 occurs upon ETS-2 reduction in prostate cancer cells, resulting in growth inhibition [45]. Furthermore, the PI3K/Akt pathway is also able to regulate cyclin D1 via GSK3Beta and Beta-catenin [46]. In colorectal cancer, beta-catenin is a direct transcriptional target of ESE-1 and down-regulating ESE-1 inhibits tumorigenic properties via beta-catenin inhibition [47]. The human cyclin D1 promoter has regulatory sites for several transcription factors, including ETS-2 and NF-KB. Notably, the ESE-1 promoter has NF-KB binding sites and, in prostate cancer cells, ESE-1 and NF-KB are involved in a positive feedback loop, whereby ESE-1 binds to ETS binding sites on the NF-KB promoter and thus induces genes that enhance the tumorigenic phenotype of LNCaP cells [48]. This raises the possibility that down-regulation of cyclin D1 is an indirect effect of ESE-1 regulating NF-KB. Nevertheless, a reduction of cyclin D1 is common to both cell lines upon ESE-1 KD, indicating that cyclin D1 is another key ESE-1 effector for controlling the transformed phenotype in both cell types.

To elucidate additional common and distinct effectors that might serve to control the transformed phenotype via ESE-1, we generated whole gene expression data. We found that knocking down ESE-1 inhibited distinct genes known to be important for tumorigenesis and maintenance of tumor initiating cells in both cell types (Supplementary Figure 7A). We next used QPCR to validate changes in expression of some these genes in both cell lines upon ESE-1 KD (Supplementary Figure 7B). We found that ESE-1 KD did indeed reduce ESE-1 mRNA in both the gene array and QPCR validation studies, in both cell types, serving as a positive control for the utility of these results. QPCR analysis verified that *CCND1, ATF3, BRAF* mRNA expression are down-regulated upon ESE-1 knockdown in BT474 cells (Supplementary Figure 7B). In SKBR3 cells, we verified that ESE-1 knockdown reduces *CCND1*, *STAT5B, JAK3, and CXCR4* mRNA expression (Supplementary Figure 7B). Notably, a reduction in cyclin D1 occurs in both cell types, underscoring the importance of ESE-1 in regulating its expression. Another key difference between these two HER2^+^ cell types is that BT474 cells are ER-positive luminal B cells and are dependent on estradiol for optimal growth. Our whole gene expression data suggest that knockdown of ESE-1 leads to inhibition of key ERalpha target genes, such as *TFF1, CCND1, RAB31, ITGBL1* and *Myb* [49] (Supplementary Figure 7A), indicating that ESE-1 inhibition affects ER dependent pathways. The other ETS transcription with a known role in hormone dependent cancers is ESE-2/Elf5. Elf5 expression opposes the action of estrogen by suppression of ERalpha and ERalpha regulated genes and contributes to tamoxifen resistance in luminal A cells. In BT474 cells, ESE-1 most likely cooperates with ERalpha to drive transformation. In SKBR3 cells, which are ER^-^, knockdown of ESE-1 specifically affects genes involved in the regulation of NF-Kb pathway, which further underscores that difference in ESE-1 mediated tumorigenicity in the two cell lines.

Finally, we show that knocking down ESE-1 significantly inhibits tumorigenesis in NOD/SCID mice (Figure 7). We were limited to BT-474 cells for this xenograft tumor study, since no other HER2+ cell lines generate tumors in immunodeficient mice. The one small nodule that did grow, albeit slowly, was undetectable by bioluminescence imaging after week 2, indicating that it had likely eventually lost both the luciferase and shESE-1 KD vectors. Consistent with this interpretation, H&E histological examination showed that the nodule was a mammary epithelial cell tumor, similar to scramble controls. Because we found a near complete inhibition in tumorigenesis, we suspected that ESE-1 plays a role in initiating tumors. In an *in vitro* tumorigenic assay, knocking down ESE-1 significantly inhibited tumorsphere formation, revealing that ESE-1 contributes to the maintenance of tumor initiating cells (Figure 8). Previous reports have established that the HER2/PI3K/Akt signaling axis is critical to proliferation and sustenance of tumor initiating cells [34, 50, 51]. In addition, the NF-KB, IL-8-CXCR1/2, JAK-STAT axes have been implicated in promoting self-renewal and differentiation of HER2^+^ breast cancer stem cells [34, 50, 51]. As mentioned above, we found that expression of some of these key genes are altered upon ESE-1 knockdown (Supplementary Figure 7). Future efforts will be directed towards finding the precise contribution of each of these genes to ESE-1 mediated tumorsphere formation.

The data presented in this study describe a central role for ESE-1 in controlling transformation in HER2^+^ cells. The fact that ESE-1 knockdown inhibits transformation properties in the face of adaptive signaling has significant implications in trastuzumab resistance. Use of mTOR inhibitors in combination with trastuzumab or lapatinib is currently underway for cancers that are refractory to the anti-ERBB2 therapy, mostly caused by aberrant PI3K/Akt/mTOR signaling. Collectively our findings suggest that ESE-1 could be central towards controlling a diverse gene repertoire other than those involved in the putative axis and therefore can be a potential therapeutic target.

## MATERIALS AND METHODS

### Cell lines and patient tumor samples

Human nontransformed MCF-10A MECs, and luminal A MCF-7, luminal B, ER^+^ HER2^+^ BT474, and HER2 subtype SKBR3 breast cancer cell lines were maintained in ATCC prescribed media [8]. Patient breast cancer tumor samples used were part of the GOBO repository, which were derived from public repositories and consists of 11 public datasets [27].

### shRNA constructs and transduction and siRNA transfection

All pLKO.1 shRNA constructs are from Open Biosystems. Oligonucleotide shESE-1_1.3 (5'-gccatgaggtactactacaaac-3') targets the ETS domain and shESE-1_1.5 (5'-gcaactacttcagtgcgatgtac-3') targets the Pointed domain. The shScr scrambled control was a gift from Dr. Bolin Liu [52]. All shRNAs were packaged into lentivirus and cells were transduced at a ratio of 1:3 of viral supernatant to media [40] . The siRNAs were custom synthesized by GE Dharmacon (Lafayette, CO) to span the target sequence of shESE-1_1.3 and shESE-1_1.5. SKBR3 and BT474 cells (600,000 cells) were transfected with 70 nM siRNA, following GE Dharmacon's protocol for siRNA transfection.

### Cell proliferation, clonogenicity and soft agar assays

Cell proliferation, clonogenicity, and soft agar assays were performed following the procedures described below. All cells were transduced with shRNAs prior to plating. For proliferation assays, cells were transduced, treated with puromycin (4ug/ml) and 5000 transduced cells/well were plated in quadruplicate in 96 well plates, and then stained with crystal violet on days 1, 3, 5 and 7. For clonogenicity studies, 3000 transduced cells were plated/well in quadruplicate in 6 well plates and stained on day 11. For soft agar assays, transduced SKBR3 and BT474 cells were suspended at 30,000 cells/well and 20,000 cells/well, respectively, and followed through day 17.

### Apoptosis and viability assay

Cells were transfected with siScr and siESE-1, and plated at 5000 cells/well in 96 well plates. After 72 hours, the Caspase 3/7 reagent (Promega, Madison, WI) was added to the cells and apoptosis assays were performed as described previously [8]. The trypan blue exclusion viability assays were performed using the Vicell. [8]

### Cell cycle analysis

DNA cell cycle analysis was done using saponin/PI staining. SKBR3 and BT474 cells were transduced with shRNA and 100,000 cells were plated in 10 cm plates, and harvested at days 8 and 13 post-transduction. BT474 cells were synchronized using 10 uM lovastatin for 24 hrs. and released with mevalonate (at 100X the lovastatin concentration). Briefly, cells were washed with PBS, pelleted and re-suspended in 1 ml of Saponin/PI stain. The cells were left overnight for 4^°^C and submitted for cell cycle analysis to the UC Cancer Center cell cycle analysis and flow cytometry core. SKBR3 cells required growth in 5% serum after transduction, for cell cycle experiments.

### Western blotting

Western blotting was performed as described previously [8]. Cells were harvested in RIPA lysis buffer (150 mM sodium chloride, 1.0% NP-40 or Triton X-100, 0.5% sodium deoxycholate, 0.1% SDS (sodium dodecyl sulphate), 50 mM Tris, pH 8.0) supplemented with protease inhibitor cocktail (Roche) and phosphatase inhibitor cocktail from Roche. Proteins were quantified using the Bio Rad DC protein assay, following which (25-50) ug of proteins were loaded on SDS PAGE gel and Western blotted. The blots were incubated with primary antibodies, anti-ESE-1,mAb 405 (1:1000; [8], anti-pAKT (Cell signaling; 1:1500), anti-AKT (Cell signaling; 1:750), anti-pMAPK (Cell signaling; 1:1000), anti- MAPK(Upstate; 1:7500), anti-HER2 (Cell signaling; 1:2000), anti-pHER2 (Cell signaling;1:1500), anti-p27 (Santa cruz;1:500), anti-cyclin D1 (Cell signaling;1:100), anti-cyclin D3 (Santa cruz; 1:500) as indicated in the figures. Anti-tubulin (Calciochem; 1:10000) was used as a loading control. HRP conjugated polyclonal goat antibodies against mouse or rabbit was used as the secondary antibody. Densitometry analysis was done using the NIH Image J software. Figure 5(A-C) are representative images of three biological replicates. The tubulin controls to which all other proteins were normalized have been provided at the bottom of blot sections as necessary.

### Immunocytochemistry (ICC)

For analysis of subcellular localization of ESE-1, 50,000 cells were plated directly on to glass cover slips in a 12 well tissue culture plate. Two days post-plating, cells were fixed with 4% paraformaldehyde (PFA) in 1X PBS for 15 minutes at room temperature, followed by three 5 min washed with 1X PBS. Cells were then permeabilized at room temperature with 0.5% Triton X-100 in 1X PBS for 10 mins, followed by three 10 min washes in 100 mM glycine in 1X PBS. Permeabilized cells were blocked in 1X PBS, 0.5% Tween-20, 10% goat serum, 0.05% bovine serum albumin blocking buffer (BSA) at RT within a moisture chamber for 1-2 h. Cells probed for ESE-1 were incubated with 1:500 antibody in blocking buffer overnight, washed three times for 10 mins in 1XPBS/0.2% BSA, and incubated for 1 hour with a Cy2 conjugated anti-mouse secondary for 1 hour in the dark. Coverslips were then washed again three times and mounted on a microscopic slide using Moviol mounting medium. DAPI counterstaining was done as necessary. To measure auto-fluorescence, cells were incubated overnight with blocking buffer alone.

### BrdU labelling and ICC

For the BrdU uptake analysis, SKBR3 and BT474 cells were transduced with shScr and shESE-1_1.3, selected with puromycin (4ug/ml), and plated in dual BD Falcon culture slides. SKBR3 cells were monitored through 3 days while BT474 cells were monitored through 8 days. Since knocking down ESE-1 decreases proliferation, 10,000 cells of shScr and 20,000 cells of shESE-1_1.3 were plated to achieve an equal representation of cell number between shScr and shESE-1_1.3 at the end point. 8 or 3 days post plating cells were pulsed with 10uM of BrdU labeling solution and incubated for 2 hours at 37°C. Post pulsing, the cells were washed in PBS two times and fixed in 80% ice cold methanol. Culture slides were then placed in preheated 2N HCL for 90 mins at 37°C, washed in distilled water for 2 mins and placed in 1M Sodium borate solution for 5 mins, and rinsed again with distilled water. Endogenous peroxidase was blocked using 3% hydrogen peroxide for 5 mins at RT. Slides were washed in PBST three times for 5 mins each and then incubated with mouse anti-BrdU at 1:50 dilutions for 1 hour at RT. A mouse IgG was used a negative control at 1:1000 dilution. Cells were rinsed in PBST three times for 5 mins and a biotinylated anti-mouse IgG was used as a secondary antibody at 1:200 dilution in RT for 2 hours. Cells were rinsed and then stained using DAB, counterstained with Haematoxylin, dehydrated in graded ethanol and mounted on microscopic slides with vectashield. For scoring purposes, any cells that had brown nuclei staining were considered positive for BrdU, irrespective of the degree of staining intensity. Ten different fields of each slides were then randomly counted in a blinded fashion. Data shown is the average of 3 biological experimental repeats.

### Tumor xenograft assay

NOD.CB17-*Prkdcscid*/NCrHsd (NOD.SCID) mice at 6 weeks of age were purchased from Harlan Laboratories (Denver, CO). The mice were implanted with estrogen pellets for continuous release of estrogen (E2) through the length of the study. Pools of BT474 cells stably expressing luciferase were transduced with shScr or shESE-1_1.3, and then selected with 4 ug/ml of puromycin overnight and plates were replaced with puromycin-free complete media the day after. Cells were harvested the following day with Trypsin/EDTA and re-suspended in 1X matrigel (BD Bioscience, San Jose, CA) at a concentration of 2x10^6^ cells/50 ul. The bilateral inguinal mammary fat pads of 7 mice were injected with cells transduced with shScr control and 8 mice were injected with cells transduced with shESE-1_1.3. Tumor growth was monitored by bioluminescence imaging using the Xenogen IVIS Lumina system (Toronto, CA), using the protocol detailed in the University of Colorado Cancer Center Animal Imaging Core Facility website.

### Tumorsphere assay

Tumorsphere assay was performed following the protocol from STEMCELL technologies. Briefly, 1000 single cell suspension of BT474 or SKBR3 were plated in low adherent plates and passaged for two generations in mammocult complete medium from STEMCELL. Tumor forming capacity was measured by counting tumorspheres greater than 75uM in diameter in the scramble control and the ESE-1 knocked down cells.

## SUPPLEMENTARY MATERIALS FIGURES



## Abbreviations

RFS: relapse free survival
DFS: disease free survival
KM: Kaplan Meier
KD: knockdown
GOBO: gene ontology based outcome
CA: constitutively active
S.A: soft agar
