# Supplementary material for: ESE-1/ELF3 mRNA expression associates with poor survival outcomes in HER2+ breast cancer patients and is critical for tumorigenesis in HER2+ breast cancer cells
Source: Oncotarget. 2017 Jun 27;8(41):69622–40. doi: 10.18632/oncotarget.18710 (PMC5642504; doi:10.18632/oncotarget.18710)
Supplement: Supplementary file 1 [file oncotarget-08-69622-s001.pdf]

ESE-1/ELF3 mRNA expression associates with poor survival outcomes in HER2+ breast cancer patients and is critical for tumorigenesis in HER2+ breast cancer cells

SUPPLEMENTARY MATERIALS

REFERENCES

1. Cerami E, Gao J, Dogrusoz U, Gross BE, Sumer SO, Aksoy BA, Jacobsen A, Byrne CJ, Heuer ML, Larsson E, Antipin Y, Reva B, Goldberg AP, et al. The cBio cancer genomics portal: an open platform for exploring multidimensional cancer genomics data. Cancer Discov. 2012; 2:401-404.
2. Gao J, Aksoy BA, Dogrusoz U, Dresdner G, Gross B, Sumer SO, Sun Y, Jacobsen A, Sinha R, Larsson E, Cerami E, Sander C, Schultz N. Integrative analysis of complex cancer genomics and clinical profiles using the cBioPortal. Sci Signal. 2013; 6:pl1.
3. Neve RM, Chin K, Fridlyand J, Yeh J, Baehner FL, Fevr T, Clark L, Bayani N, Coppe JP, Tong F, Speed T, Spellman PT, DeVries S, et al. A collection of breast cancer cell lines for the study of functionally distinct cancer subtypes. Cancer Cell. 2006; 10:515-527.
4. Rhodes DR, Kalyana-Sundaram S, Mahavisno V, Varambally R, Yu J, Briggs BB, Barrette TR, Anstet MJ, Kincead-Beal C, Kulkarni P, Varambally S, Ghosh D, Chinnaiyan AM, et al. Oncomine 3.0: genes, pathways, and networks in a collection of 18,000 cancer gene expression profiles. Neoplasia. 2007; 9:166-180.

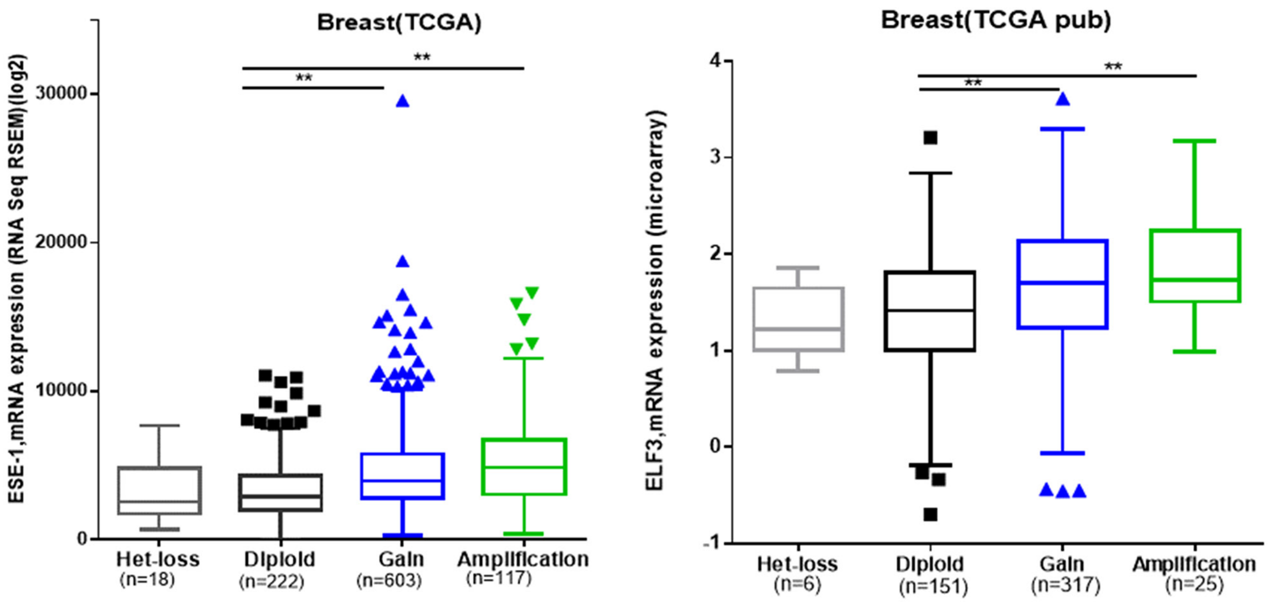

Supplementary Figure 1: Gained and amplified ESE-1/ELF3 CNV in breast cancer associates significantly ($P < 0.01$, Mann-Whitney test), with upregulated mRNA expression (obtained from RNA seq data). Het-loss represents loss of heterozygosity. Plots have been constructed using the Breast (TCGA) and Breast (TCGA pub) datasets from the cBioPortal for cancer genomics [1,2]. All TCGA data here are in whole or in part based upon data generated by the TCGA Research Network: http://cancergenome.nih.gov/.

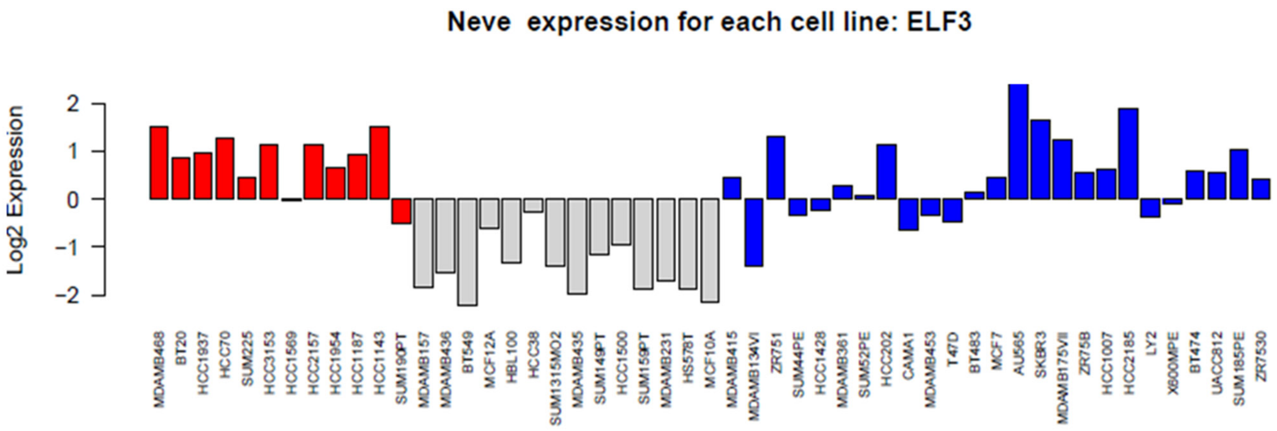

Supplementary Figure 2: Normalized ESE-1 gene expression in the 51 immortalized cell lines generated by GOBO analysis. Cell lines have been classified based on the gene expression profiles into triple negative (Red bars), basal (Grey bars), and luminal (Blue bars) by Neve et al [3]. The y-axis is the log2 mRNA expression of ESE-1 and the x-axis is the number of cell lines. All of the $HER2^+$ cell lines associate with the luminal, based on their gene expression profile, and therefore were color coded as Blue bars.

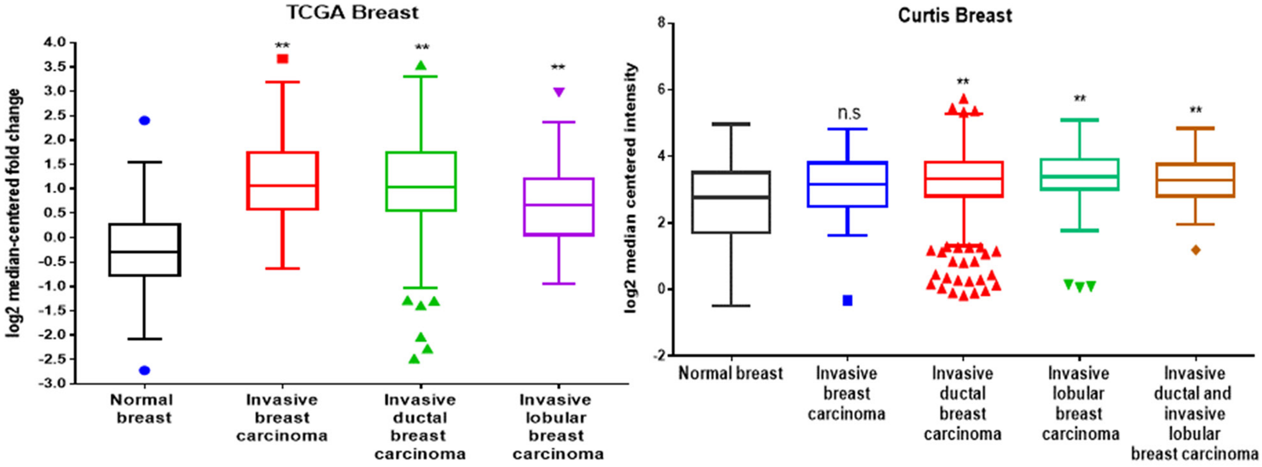

Supplementary Figure 3: The ESE-1/ELF3 mRNA level (log2 median centered intensities obtained from microarray) is significantly upregulated in invasive breast carcinomas (Anova P value <.0001) compared to the normal breast. Plot has been generated using the TCGA Breast data from Oncomine [4].

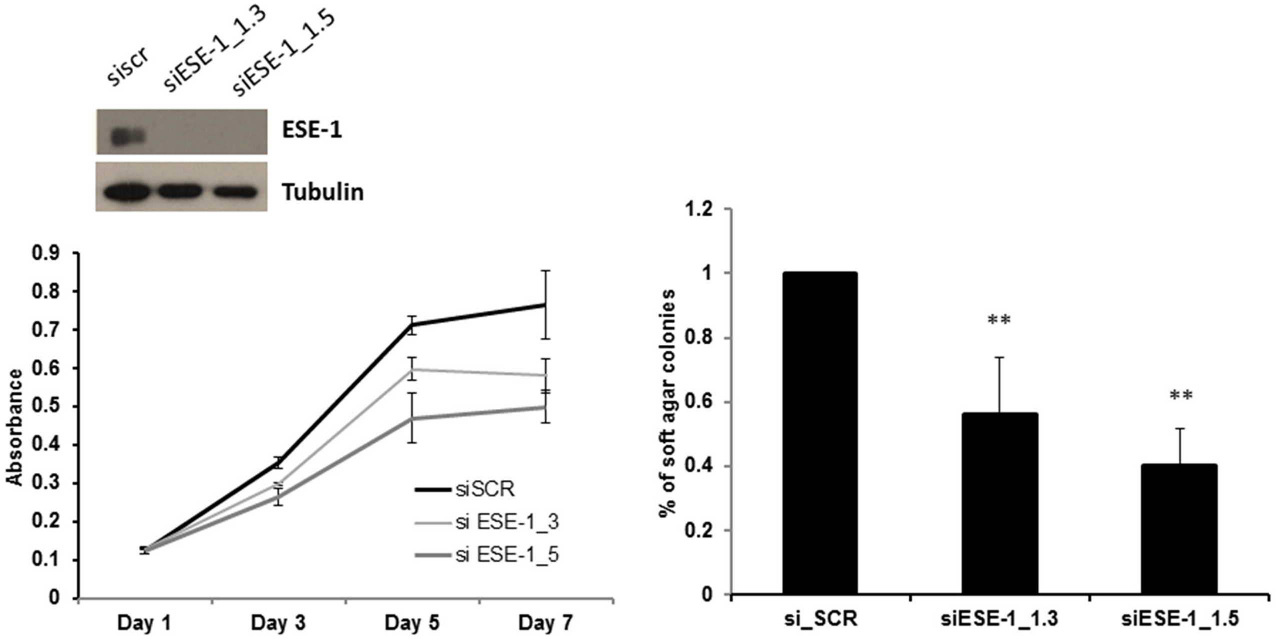

Supplementary Figure 4: Biological response is directly related to the level of ESE-1 knockdown achieved. The Western blot shows that ESE-1 has been almost completely inhibited using siESE-1_1.3 and siESE-1_1.5 at day 2 post siRNA transfection. Knocking down ESE-1 inhibits cell growth and soft agar colony formation. Of note, knockdown with siESE-1_1.5 elicits a 50% decrease in soft agar colony formation, whereas knockdown with shESE-1_1.5 resulted in a 20% decrease in soft agar colony numbers at day 19 post transfection. We attribute this difference to the more complete inhibition of ESE-1 that was achieved using siESE-1_1.5.

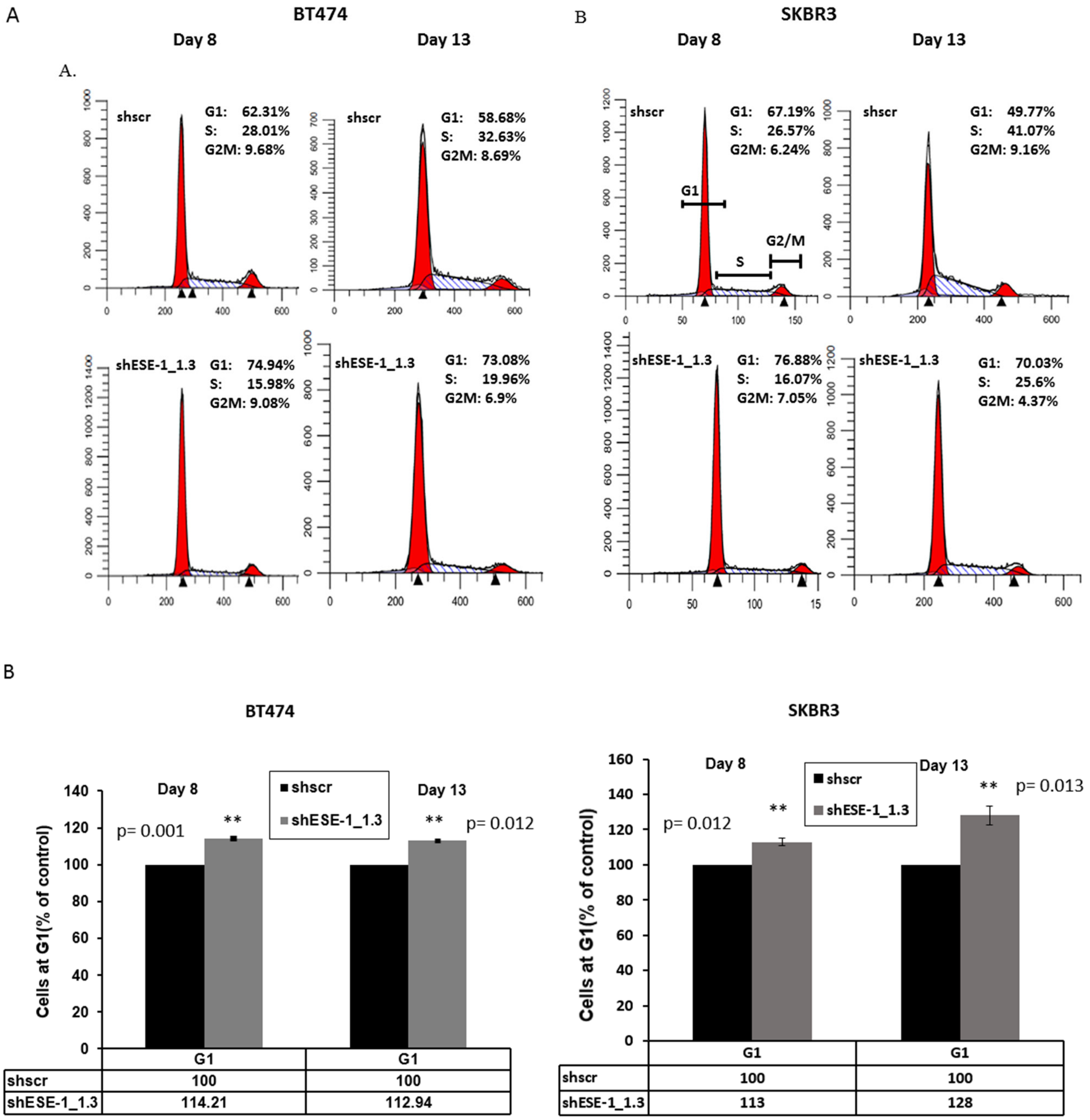

BT474

	G1	G1
shscr	100	100
shESE-1_1.3	114.21	112.94

SKBR3

	G1	G1
shscr	100	100
shESE-1_1.3	113	128

Supplementary Figure 5: (A) Flow cytometry cell cycle analysis. Each of the figures at day 8 and day 13 is one of three biological repeats. **(B)** Statistical analysis of all three cell cycle replicates for percent of cells accrued at G1 upon ESE-1 knockdown. Percent of cells accrued at G1 for scramble controls in each of the biological replicates was set as 100. Cells accrued at G1 in the knockdowns was calculated as a percent of the scramble control. Accrual of cells at G1 increases on average from 113% at day 8 to 128% at day 13 in SKBR3 cells. Accrual of SKBR3 cells is significant to p values of 0.012 at Day 8 and to p values of 0.013 at day 13.Accrual of BT474 cells at G1 is significant to p values of 0.001 at day 8 and 0.012 at day 13. In BT474 cells, on average there is little or no change in accrual of cells going from day 8 to day 13.

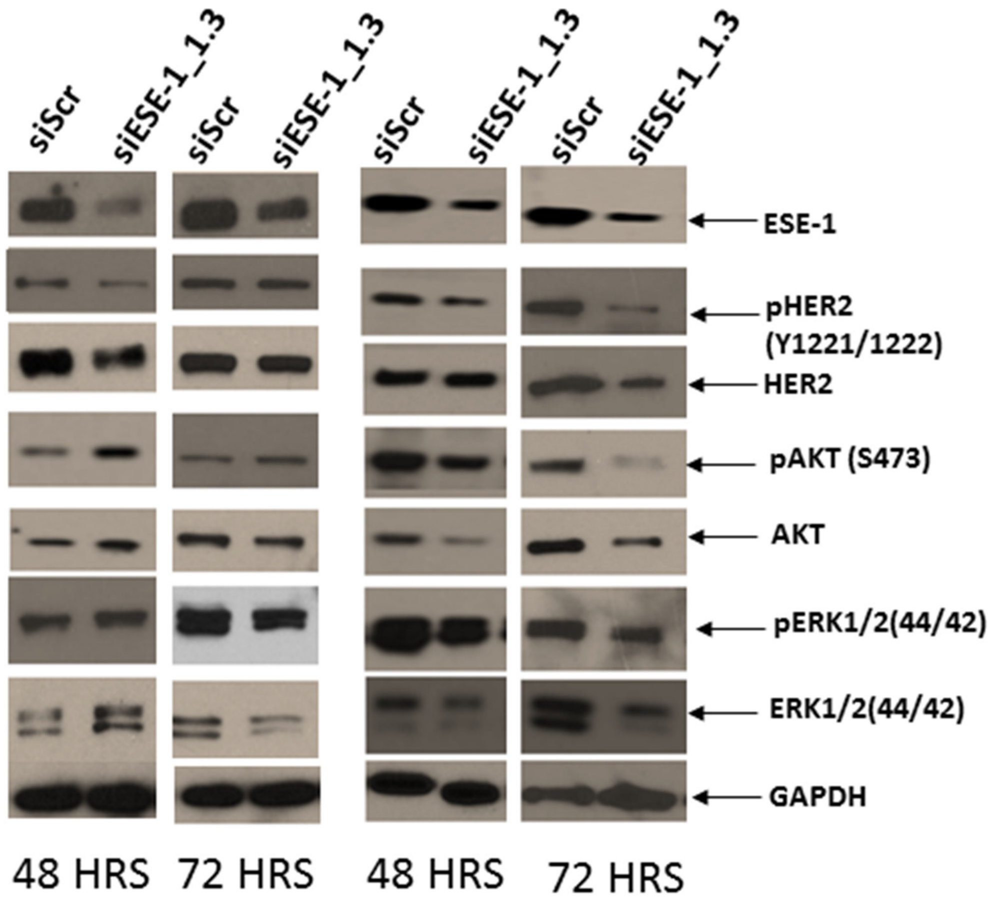

Supplementary Figure 6: Western blotting for signaling proteins upon transient knockdown of ESE-1 using siSCR and siESE_1.3 at 48 hours and 72 hours post-transfection. BT474 cells showed a decrease in ESE-1 and HER2 expression, but slight increase in Akt and pAktS473 at 48-hrs post-transfection, whereas the decrease in these proteins was less robust at 72-hrs. The SKBR3 cells evinced a decrease in ESE-1, pHER2, Akt and pAktS473 at both 48 and 72 hrs post-transfection.

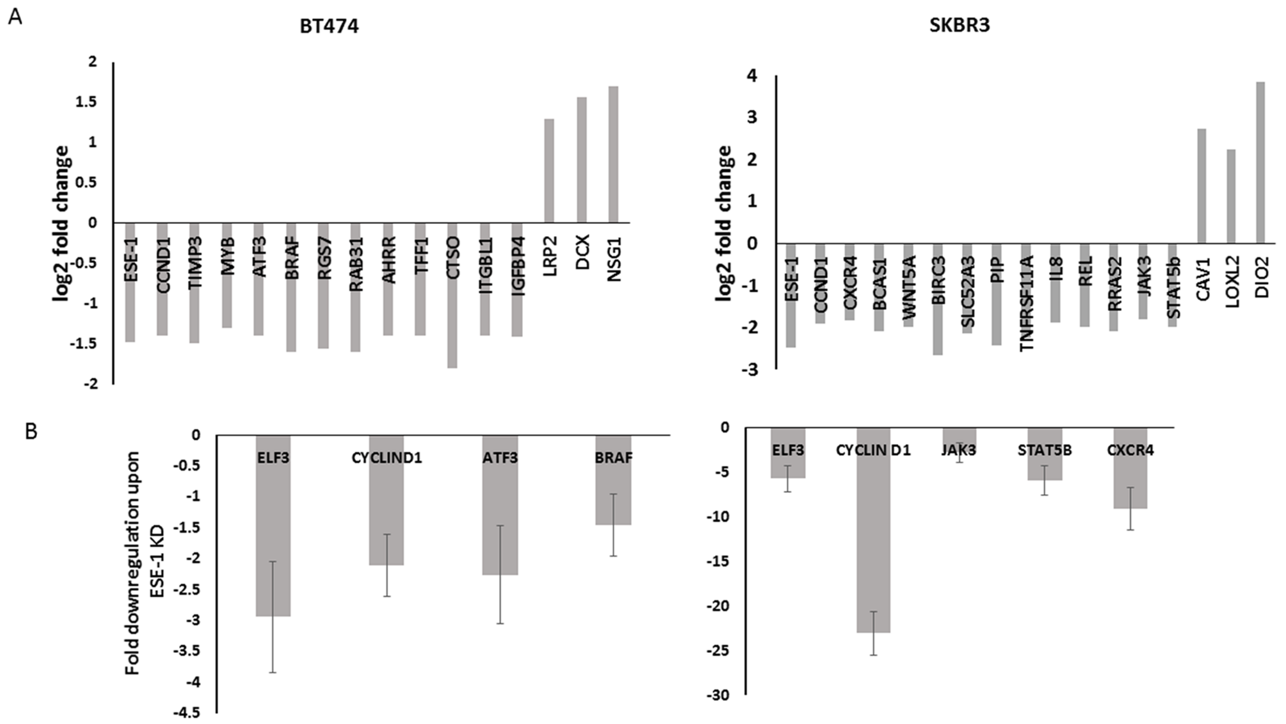

Supplementary Figure 7: (A) Whole gene expression analysis of BT474 and SKBR3 cells were done upon transient knockdown of ESE-1 using two distinct siRNAs, ESE-1_1.3 and ESE-1_1.5, at 48 hours post-transfection. The microarray platform was Affymetrix HG-U133plusII. One replicate of each cell line was used. Normalization of the raw data was done using the Affy package. The cutoff used for analysis of up and downregulated gene expression in each cell type was based of the fold downregulation observed in ESE-1 gene expression. In BT474, the cutoff was set at ≥ 1.4 and in SKBR3 cells the cutoff was set at ≥1.9. **(B)** QPCR validation of selective genes from the microarray. Genes for QPCR validation were chosen based on their relevancy in $HER2^+$ breast cancer. Relative quantitation was performed using the C^t values and duplicate independent reactions, each with two technical replicates, to reach the average C^t for genes from the knockdown samples and the scramble control. GAPDH was used as the reference gene for calculating the ΔC^t. Figure S8B shows fold-change in expression for genes that were downregulated in the knockdown samples compared to the scramble control calculated by the $\Delta\Delta C^t$ method. Error bars represent the range of the $\Delta\Delta$ C^t values incorporated into the fold change.
